# A pattern-growth approach for mining maximal fault-tolerant frequent itemsets

**DOI:** 10.1038/s41598-026-44941-3

**Published:** 2026-03-23

**Authors:** Shariq Bashir

**Affiliations:** College of Computer and Information Sciences, Imam Mohammad Ibn Saud Islamic University (IMSIU), Riyadh, Saudi Arabia

**Keywords:** Engineering, Mathematics and computing

## Abstract

Mining fault-tolerant (FT) frequent itemsets in noisy datasets is more challenging than conventional frequent itemset mining due to the high cost of evaluating fault-tolerance conditions. Consequently, mining maximal fault-tolerant frequent itemsets (FT-MFIs) is particularly important, as they provide a concise representation of all frequent patterns while eliminating redundancy, which is crucial in noisy datasets where error tolerance must be considered. Existing approaches for mining FT-MFIs are predominantly based on Apriori-style candidate generation and test, which suffer from exponential candidate growth, multiple dataset scans, and limited scalability. As a novel contribution, this work is the first to propose a pattern-growth framework for mining FT-MFIs. We introduce an algorithm that constructs a fault-tolerant FP-tree (FT-FP-tree) to compress transactions with common prefixes and evaluate tolerance conditions in a single pass. Additionally, new techniques for transaction mapping and conditional pattern extraction enhance the efficiency and scalability of the mining process. Experimental results on benchmark datasets demonstrate that the proposed approach achieves substantial reductions in execution time compared to all existing algorithms, while also showing improved memory efficiency relative to other pattern-growth based algorithms. This establishes pattern growth as a practical and scalable solution for fault-tolerant frequent itemset discovery.

## Introduction

Frequent itemset mining (FIM) has long been considered one of the most fundamental problems in the broader domain of data mining ^1^. The discovery of frequently co-occurring items in large datasets forms the foundation for numerous data-driven applications across a variety of fields ^2^. For instance, FIM enables effective clustering of documents by identifying groups of terms that appear together in textual corpora^3–5^. In social network analysis, itemset mining supports the detection of community structures and interaction patterns among users^6–8^. Similarly, in commercial environments, market basket analysis remains one of the most well-known applications, where co-purchased products are extracted to guide cross-selling and recommendation systems^9,10^. Beyond business, FIM has also been applied in several other domains. In fraud detection, it helps uncover suspicious transaction patterns^11–13^. In bioinformatics, it is used to identify recurring biological markers or gene interactions^14–17^. In web mining, FIM supports the analysis of usage logs to capture user navigation behavior^18–20^.

The formalization of association rule learning, introduced in the foundational work of^21^, placed FIM at the core of data mining research. Since then, an extensive body of work has emerged that has focused on designing efficient algorithms for discovering frequent itemsets (patterns) in transactional datasets^22–34^. Despite the maturity of this area, traditional FIM suffers from a key weakness: It assumes that frequent itemsets must be identified through exact matches within the database.

### Challenges of conventional FIM

Reliance on exact matching restricts the utility of conventional FIM when applied to noisy or incomplete datasets. Real-world datasets rarely conform to clean and exact patterns due to errors, omissions, or inconsistencies in recording processes^35–38^. Consequently, itemsets that should intuitively be considered frequent may be overlooked because they are not perfectly present across transactions. Furthermore, the choice of support threshold has a critical impact on the output. When the threshold is set too high, the algorithm produces only a small number of very short itemsets. In contrast, lowering the threshold leads to an explosion of trivial itemsets, especially those of length two or three^39–41^. Both extremes reduce the practical usefulness of mined itemsets.

### Fault-tolerant (FT) frequent itemsets mining

To overcome these issues, the concept of *fault-tolerant* (FT) frequent itemsets was proposed by^35^. The key idea is to relax the rigid requirement of exact matching by introducing a tolerance factor, $\delta$, that allows for the presence of noise or missing items. Under this model:

1. An itemset *X* qualifies as an FT frequent itemset if it is supported by at least $(min\_sup^{\delta })$ FT transactions.
2. A transaction is said to be an FT transaction of *X* if it contains at least $(|{X}|-\delta )$ items from *X*.
3. Additionally, each item in *X* must occur in at least $(item\_sup^{\delta })$ FT transactions of *X*, ensuring that tolerance does not compromise the reliability of individual items.

This formulation allows the discovery of meaningful patterns even when the dataset is imperfect. The FT parameter, $\delta$, determines how many items of an itemset *X* are absent in a transaction while still considering that transaction as supporting *X*. For an itemset of length *X*, at least $({X} - \delta )$ items must appear together to satisfy the FT condition. A smaller $\delta$ (for example, $\delta = 0$ or 1) implies that only transactions with almost complete presence of all items in *X* are counted, corresponding to low tolerance for noise. As $\delta$ increases, the algorithm becomes more flexible and includes transactions where more items are missing. For instance, if ${X} = \{a, b, c, d\}$ and $\delta = 1$, a transaction containing any three of these items (e.g., $\{a, b, c\}$) still contributes to the support of *X*. When $\delta = 2$, even transactions containing only two items from *X* are counted. However, if $\delta$ approaches |*X*| (for example, $\delta = 3$ for a four itemset), almost any transaction containing at least one of the items in *X* will qualify as a FT occurrence, reducing the discriminative power of the measure. Therefore, $\delta$ should be chosen carefully according to the expected noise level in the data to balance completeness and specificity. In practical settings, smaller $\delta$ values are suitable for relatively clean datasets, whereas larger $\delta$ values are beneficial when data contain omissions or measurement errors. Table 1 summarizes how different $\delta$ values influence both the minimum required items and the discriminative nature of the patterns. Increasing $\delta$ relaxes the FT constraint, allowing more partially matched itemsets to be considered frequent. Although this expands the search space, it reduces the discriminative power of the resulting patterns because fewer items are required to co-occur. Smaller values of $\delta$ enforce stricter matching, producing more specific and more discriminative maximal FT frequent itemsets.

**Table 1:** Effect of $\delta$ on required items and discriminative power. *X* is the length of itemset.

$\delta$	Minimum required items $({X} - \delta )$	Tolerance to missing items	Discriminative power
0	*X*	None	Very high
1	${X} - 1$	Low	High
2	${X} - 2$	Moderate	Medium
3	${X} - 3$	High	Low
Large $\delta$	Small	Very high	Very low

**Table 2:** Sample e-commerce transactional dataset showing items purchased and the resulting frequent items across transactions with a minimum support threshold of $\texttt {min\_sup}=3$.

TID	Items	Frequent items
10	*Keyboard, Headphones, Smartphone, Mouse*	*Laptop, Mouse, Headphones, Keyboard*
20	*Headphones, Laptop, Mouse*	*Laptop, Mouse, Headphones*
30	*Laptop Stand, Smartphone, Mouse*	*Mouse, Laptop Stand, Smartphone*
40	*Laptop, USB Adapter*	*Laptop*
50	*Laptop Stand, Keyboard, Headphones, Laptop*	*Laptop, Laptop Stand, Headphones, Keyboard*
60	*Laptop Stand, Laptop, Mouse*	*Laptop, Mouse, Laptop Stand*
70	*Keyboard, Laptop Stand, Smartphone, Mouse*	*Mouse, Laptop Stand, Smartphone, Keyboard*
80	*Smartphone, Mouse Pad, Power Bank*	*Smartphone*
90	*Smartphone, USB Cable, SD Card*	*Smartphone*

To better illustrate the practical interpretation of FT frequent itemsets, consider the e-commerce transaction dataset shown in Table 2, consisting of nine shopping-cart transactions. With a minimum support threshold of $\texttt {min\_sup}=3$, conventional exact frequent itemset mining identifies only short itemsets of length at most two (such as *Laptop+Mouse* or *Laptop+Headphones*). These short itemsets provide limited insight because real customers frequently purchase product bundles that may vary slightly from one transaction to another. When fault tolerance is introduced with $\delta = 1$, longer and more meaningful purchasing patterns emerge. For example, the itemset ${X}=\{\textit{Laptop},\ \textit{Mouse},\ \textit{Headphones},\ \textit{Keyboard}\}$ forms a FT frequent itemset of length four with FT-support equal to three. Transactions 10, 20, and 50 each contain at least three of these items, satisfying the FT condition that allows one item to be missing. Furthermore, each item in *X* appears in at least two distinct transactions, meeting the individual FT-occurrence requirement. Under exact matching, *X* would not be considered frequent because no transaction contains all four items simultaneously. Under FT support, however, *X* becomes a valid and highly informative FT frequent itemset, capturing a realistic customer purchasing pattern (a typical *laptop workstation bundle*). This example demonstrates that fault-tolerant mining uncovers longer, behaviorally meaningful itemsets that traditional exact FIM would entirely miss, thereby revealing richer purchasing structures in e-commerce environments.

FT frequent itemset mining is highly relevant for many real world applications where transactional records are incomplete, noisy, or imprecise. Examples include retail sales data, sensor logs, Internet of Things (IoT), web clickstreams, healthcare records ^42^, and industrial monitoring. In these domains, missing entries, intermittent sensor failures, anonymized identifiers, and recording errors are common, so methods that tolerate small amounts of noise while still extracting reliable patterns are particularly useful ^43,44^. In retail, FT itemset mining can reveal robust co-purchase relationships even when some barcodes are missing or transactions are partially recorded. Such patterns support assortment planning, cross-selling, and targeted promotions by identifying groups of products that are frequently bought together despite occasional recording errors. In sensor networks and IoT deployments, FT mining can detect recurring combinations of sensor readings that indicate normal operating modes or emerging faults, even when individual sensors drop out or report noisy values. For example, a recurring pattern of temperature, vibration, and current readings can signal an incipient fault of the equipment, even if one sensor intermittently fails. In web and mobile analytics, FT itemset mining can identify stable navigation patterns or frequent sequences of actions even when some events are lost during logging or anonymized. In healthcare and clinical data, FT patterns can help find common co-occurrences of symptoms, medications, or lab results across incomplete electronic health records, supporting cohort discovery and hypothesis generation. In cyber security and fraud detection, FT patterns expose typical combinations of events that precede attacks or fraudulent transactions while tolerating gaps in noisy logs ^11–13^ (Table 3).

**Table 3:** Example of a transactional dataset with frequent items across transactions.

TID	Items	(Ordered) frequent items
10	*c , d , e , g*	*f , g , d , c*
20	*d , f , g*	*f , g , d*
30	*b , e , g*	*g , b , e*
40	*f , l*	*f*
50	*b , c , d , f*	*f , b , d , c*
60	*b , f , g*	*f , g , b*
70	*c , b , e, g*	*g , b , e, c*
80	*e , h , j*	*e*
90	*e , i , k*	*e*

### Limitations of Apriori-based FT frequent itemset mining and main contribution

Early algorithms for mining FT frequent itemsets extended the Apriori principle, employing candidate generation and iterative testing to construct larger itemsets from smaller ones. While straightforward, this strategy faces well-known scalability issues. When support thresholds are low, the number of generated candidates grows exponentially, leading to excessive computational overhead^45–47^.

To mitigate these challenges, researchers have advocated mining only long or maximal FT frequent itemsets (FT-MFIs), rather than enumerating the complete set^45,48^. The appeal of this approach is twofold: (i) the number of FT-MFIs is significantly smaller than the full set of FT frequent itemsets, and (ii) FT-MFIs can still serve as a condensed representation from which all FT frequent itemsets may be derived in a post-processing step. Once derived, their supports can be computed efficiently through a single dataset scan. Nonetheless, the existing FT-MFI mining algorithms remain rooted in the Apriori paradigm. Their reliance on exhaustive candidate generation and multiple dataset scans significantly hampers performance due to following reasons.

- *Apriori-based* algorithms attempt to mine the complete set of FT-MFIs by generating and testing candidate itemsets. The critical weakness of this approach lies in the exponential explosion of candidates when the dataset is large. For example, if a dataset contains 300 frequent items, the *Apriori-based* approach theoretically generates and evaluates all $2^{300}$ candidate itemsets, which is computationally infeasible in practice.
- The search strategy used by *Apriori-based* algorithms is top-down, meaning that to identify a frequent itemset *X*, the algorithm must first generate and test all $2^{|{X}|}$ subsets of *X*. This exhaustive enumeration becomes highly inefficient when datasets contain a large number of single frequent items, as the number of subsets grows exponentially with itemset size.
- To validate the FT conditions for each candidate itemset, the *Apriori-based* algorithm requires multiple scans of the entire dataset. Each scan increases the computational cost, and when the number of candidates is exponential, these repeated scans make Apriori impractical for large-scale or sparse datasets.

To address these limitations, this article introduces a novel algorithm called **FT-MFI-PG**, which mines fault-tolerant maximal frequent itemsets (FT-MFIs) using a combination of *pattern-growth* and the *FP-tree* structure ^2,49^. Mining FT-MFIs is more challenging than traditional maximal frequent itemset mining ^27^. This is because the algorithm must handle additional complexity introduced by fault-tolerance. It needs to maintain and update fault-tolerant transactions (FT transactions) that partially satisfy itemsets. Additionally, the support computation must account for both exact and partial matches. The pattern-growth paradigm is particularly well suited for this task, as it combines transaction compression with localized evaluation of FT conditions. Unlike candidate generation and test approach (Apriori), which require repeated dataset scans, FT-MFI-PG constructs a compact FP-tree that merges transactions with common prefixes. This allows partially matching transactions, which may arise due to noise or missing items, to be efficiently mapped onto shared branches while preserving fault-tolerance information through the FT-FP-tree and FT conditional pattern tables (FT-CP-Tables). The divide-and-conquer strategy of pattern-growth recursively partitions the dataset into smaller conditional subsets, enabling the mining process to focus only on feasible itemsets. In addition, FT-MFI-PG prunes infeasible itemset combinations early in the search, reducing computational overhead and improving scalability compared to conventional approaches ^22,49,50^.

The mining process of FT-MFI-PG can be summarized as follows:

1. In the first scan of the dataset, all one-length frequent items are identified, while items with support below $(item\_sup^{\delta })$ are discarded.
2. A second scan maps transactions onto the FP-tree. Frequent items are stored as nodes, and transactions are mapped onto the branches. If transactions share a common prefix, the corresponding items share branches in the FP-tree. The frequency (support) of shared subsets is stored in the nodes, which results in a compact dataset representation.
3. Using depth first search, the pattern-growth explores FT-MFIs directly from the FP-tree. Supersets of an itemset are generated by constructing conditional pattern bases from FP-tree branches. Frequent items from these conditional patterns recursively build child FP-trees, from which larger FT-MFIs are discovered.

Through this recursive process, FT-MFI-PG efficiently enumerates the complete set of FT-MFIs without the exponential candidate generation and test overhead of Apriori. We conducted experiments on multiple benchmark datasets to evaluate FT-MFI-PG against existing algorithms. The results demonstrate that FT-MFI-PG mines the complete set of FT-MFIs with significantly less computational time while maintaining correctness.

The remainder of this article is organized as follows. “Related work” section provides an extensive review of related work on mining maximal and all FT frequent itemsets, highlighting differences between the proposed algorithm and existing algorithms. “Mining maximal fault-tolerant (FT) frequent itemsets: pattern-growth methodology” section introduces the mapping of FT transactions onto the FP-tree and details the integration of pattern growth for mining FT-MFIs. “Correctness and completeness of FT-MFI-PG” section presents the correctness and completeness of FT-MFI-PG. “Pruning infrequent FT-MFIs” section discusses heuristics for pruning infrequent itemsets to reduce search space. “Experiments” section describes the benchmark datasets and presents a performance comparison of FT-MFI-PG with alternative algorithms. Finally, “Conclusion” section summarizes the key findings and contributions of proposed algorithm.

## Related work

The problem of mining fault-tolerant maximal frequent itemsets (FT-MFIs) has received limited attention, and only a few algorithms have been proposed to address it. Early research efforts predominantly adopted an Apriori-style search strategy ^48^. Apriori-based algorithms rely on the well-known anti-monotonic property: if an itemset is infrequent, all of its supersets must also be infrequent. This heuristic effectively prunes a large portion of the search space, thereby reducing the computational overhead associated with frequent itemset mining. However, the Apriori framework generates a vast number of candidate itemsets, including many that may never occur in the transaction dataset. Each set of candidates must then be validated through repeated dataset scans, which significantly increases computational cost, especially in noisy datasets where fault tolerance must be incorporated.

^35^ introduced one of the earliest algorithms in this category, namely FT-Apriori. This algorithm adopts a top-down, level-wise approach, where larger candidate itemsets are generated by extending previously discovered FT frequent itemsets. An itemset *X* is considered FT frequent if its minimum support in the noisy dataset remains above the specified tolerance threshold ($min\_sup^\delta$). The algorithm begins with single items, iteratively combining them to form larger candidates while pruning those that fail the support test. Although conceptually simple and complete, FT-Apriori suffers from two main drawbacks: (i) the number of candidates grows exponentially when many frequent single items exist, and (ii) the algorithm requires multiple full dataset scans to validate FT conditions. In practice, this translates into high time complexity, since it must enumerate and evaluate all possible subsets of an itemset before confirming its frequency status. Consequently, FT-Apriori is not well suited for large-scale or high dimensional (sparse) datasets.

To overcome the inefficiency of repeated dataset scans, researchers later investigated bit-vector projection techniques.^48^ and^45^ proposed algorithms that encode each frequent item as a bit-vector representing its occurrence across all transactions. With this structure, dataset scanning is performed only once, and subsequent support counting is achieved through efficient bitwise operations. Candidate generation still follows the Apriori-style level-wise extension, but support computations are faster, since combining two bit-vectors via logical operations directly yields the occurrence of their joint itemset. While this approach significantly reduces support counting overhead, the exponential growth of candidate itemsets persists, and therefore, scalability remains a challenge.

In response to the limitations of candidate generation, ^47,51^ explored a pattern-growth paradigm for FT itemset mining. Instead of generating and testing a large number of candidate itemsets, pattern-growth algorithms recursively project the dataset into smaller conditional sub-datasets and build prefix trees (FP-trees) to directly grow frequent patterns. In the proposed method, multiple FP-trees are constructed to represent different combinations of item presence and absence. For instance, when mining supersets of (*ab*) with $\delta =1$, four FP-trees are needed: one for transactions containing both *a* and *b*, one for those containing *a* but not *b*, one for those containing *b* but not *a*, and one for those missing both. While this strategy avoids redundant candidate generation, its main drawback lies in memory consumption and redundancy: even transactions that share many common items are mapped across multiple FP-trees. As a result, the algorithm struggles with scalability and is impractical for large datasets. The proposed approach addresses this shortcoming by designing a single FP-tree structure that accommodates transactions with varying numbers of missing items, thereby reducing both memory usage and computational complexity. Moreover, existing pattern-growth approaches attempt to mine the complete set of FT frequent itemsets, which can still be exponential in number and often include many small or trivial patterns ^52^. In contrast, the proposed approach focuses on maximal FT frequent itemsets, offering a far more compact and informative representation that avoids redundancy and provides greater practical utility.

Beyond FT itemset mining, related research has also investigated approximate frequent itemset mining, where the goal is to tolerate errors or noise in the data. For example, ^53^ introduced proportional approximate frequent itemsets (AFIs), where the degree of tolerance scales with the size of the itemset. Similarly, ^54^ studied the discovery of high utility approximate itemsets in noisy datasets. These approaches differ from FT itemset mining in that their tolerance models are proportional to itemset length, leading to distinct sets of itemsets compared to those obtained with fault-tolerant definitions. Applications of proportional AFIs have been reported in domains such as bioinformatics^15^, and various heuristics have been developed to accelerate their discovery^46,55^. However, existing AFI methods are predominantly Apriori-based and therefore suffer from inherent limitations, including exponential candidate explosion and high processing costs.

### GPU-based frequent itemset mining trends

Recent trends in frequent itemset mining research have increasingly focused on accelerating the mining process using GPU-based architectures. Parallel frequent itemsets mining using distributed GPUs ^56^ presents a distributed multi-GPU framework in which the database is partitioned across multiple GPU devices to accelerate Apriori-style and level-wise counting operations ^57,58^. The primary objective of this approach is to maximize throughput by mapping support-counting tasks to massively parallel GPU kernels while managing coordinated data exchange between devices. A Parallel FP-Growth Algorithm Based on GPU extends the FP-growth paradigm to GPU environments ^59^. This approach reorganizes transactions and conditional pattern bases into GPU-compatible structures, enabling parallel construction of conditional FP-trees and mitigating the candidate-generation overhead commonly associated with Apriori-based techniques. Its design emphasizes SIMD friendly memory layouts and kernel orchestration to efficiently parallelize FP-growth.

GMiner ^60^ introduces a GPU-optimized enumeration strategy for frequent itemset mining on large scale datasets. It focuses on balancing computational load across GPU cores, reducing CPU–GPU transfer overhead, and restructuring enumeration tasks to better fit GPU execution models. This allows large datasets to be processed effectively on one or more GPU devices. GMiner++ ^61^ further enhances GMiner by reducing redundant computations and refining memory layout designs for improved utilization of multi-GPU architectures. It also reduces repeated intersection operations, resulting in improved performance when mining exact frequent itemsets. Although these GPU-based algorithms represent the latest developments in accelerating frequent itemset mining, they differ from the proposed FT-MFI-PG algorithm in several key aspects. First, all GPU-based algorithms enumerate all frequent itemsets. In contrast, the proposed FT-MFI-PG focuses on mining maximal frequent itemsets, which represent only the most specific and non-redundant itemsets. Second, the GPU-based algorithms operate under exact matching semantics, whereas the FT-MFI-PG mines fault-tolerant (FT) frequent itemsets that allow up to a specified number of missing items per occurrence. Third, the existing algorithms are designed for GPU architectures, while the proposed FT-MFI-PG is CPU-based; however, future extensions is possible to explore GPU-accelerated FT-MFI mining to further enhance performance. The Table 4 summarizes the key conceptual differences between the FT-MFI-PG and the current GPU-based frequent itemset mining algorithms.

**Table 4:** Conceptual comparison between FT-MFI-PG and modern GPU-based FIM algorithms.

Algorithm	Exact/FT	All/maximal	Paradigm	GPU?
FT-MFI-PG (this work)	FT	Maximal	Pattern-growth (FT-FP-tree)	No
Parallel FIM on distributed GPUs ^56^	Exact	All	Apriori / counting	Yes
Parallel FP-Growth on GPU ^59^	Exact	All	FP-growth	Yes
GMiner ^60^	Exact	All	Enumeration	Yes
GMiner++ ^61^	Exact	All	Enhanced enumeration	Yes

## Mining maximal fault-tolerant (FT) frequent itemsets: pattern-growth methodology

The core of the proposed approach relies on the pattern-growth paradigm, which is a divide-and-conquer strategy for mining frequent itemsets without the need for exhaustive candidate generation. Unlike Apriori-style approaches, pattern-growth uses a compact data structure known as the FP-tree (Frequent Pattern Tree) ^22,49,50^. The FP-tree is specifically designed to compress a large transactional dataset into a highly efficient representation that preserves itemset association information. In this structure, each transaction is projected onto a path in the tree, where nodes represent items and branches correspond to entire transactions. When multiple transactions share common prefixes, their overlapping parts are stored on the same branch, and the frequency of the shared prefix is aggregated at the corresponding nodes. This compression significantly reduces redundancy, especially for dense datasets, and provides a powerful foundation for mining longer frequent itemsets.

Once the FP-tree is constructed, the pattern-growth recursively explores conditional patterns to discover maximal FT frequent itemsets (FT-MFIs). For a given itemset, its supersets are identified by extracting the conditional patterns from the FP-tree branches in which the itemset occurs. These conditional patterns are then used to generate smaller, conditional FP-trees. Each conditional tree preserves only the transactions relevant to the itemset under consideration. By recursively applying this process, the algorithm expands itemsets in a depth-first manner, enabling efficient discovery of FT-MFIs at successive levels. To facilitate traversal of the FP-tree, each occurrence of an item is connected through a linked list, and the head pointers of these lists are stored in a header table. The header table acts as an index, allowing the algorithm to quickly locate and traverse all nodes of a given item across the FP-tree. This structure plays a critical role in ensuring that candidate generation remains efficient and that the recursive construction of conditional FP-trees can be carried out without repeated scans of the original dataset.

One of the most significant advantages of the FP-tree structure is its ability to eliminate irrelevant candidate itemsets early in the mining process. Only itemsets that actually occur in the conditional patterns are expanded, while those absent from the dataset are naturally pruned. Moreover, the evaluation of FT conditions, such as the minimum support of itemsets and the item-level support thresholds can be computed directly from the conditional patterns without costly additional scans.

### Example

To illustrate how the proposed approach constructs the initial (global) FP-tree, consider the sample transactional dataset shown in Table 3. For this example, the fault-tolerant parameter is set to $\delta =2$, while the thresholds for the minimum support and minimum item support are specified as $min\_sup^{\delta }=3$ and $item\_sup^{\delta }=2$, respectively. During the initial pass over the dataset, the algorithm filters out all items that do not meet the $item\_sup^{\delta }$ requirement. This pruning step eliminates infrequent items early in the process, ensuring that only potentially useful items contribute to subsequent FP-tree construction ^35^. After this filtering stage, the resulting set of frequent items, along with their corresponding support counts, is given as $\langle (f:5), (g:5), (b:4), (e:4), (d:3), (c:3) \rangle$.

**Fig. 1:**
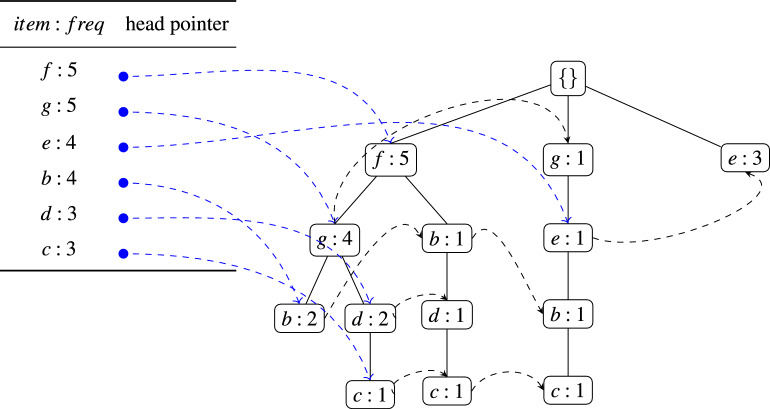
Resulting global FP-tree built from the full set of transactions.

Once the frequent items have been identified, the transactions are reorganized by ordering their items according to the global frequency of occurrence, starting with the most frequent. This reordering step is critical, as it maximizes prefix sharing in the FP-tree, thereby improving both compression efficiency and mining performance. The algorithm then performs a second pass over the dataset to construct the FP-tree. Each transaction is projected onto a path in the tree, and overlapping prefixes between transactions are merged into shared branches. For instance, when two or more transactions begin with the same sequence of items, this common prefix is stored only once in the FP-tree, and its frequency count is updated accordingly. The resulting FP-tree serves as a compact representation of the dataset, where nodes capture item frequencies and branches capture transactional structure. The constructed (global) FP-tree for the dataset in Table 3 is depicted in Fig. 1, which clearly illustrates how transaction compression and prefix sharing are achieved through the FP-tree representation.

**Table 5:** Conditional patterns of itemset (*dc*) derived from the FP-tree and FT patterns.

Item	Conditional patterns	FT conditional patterns
*c*	*fgdc* : 1	($\langle \langle fg\rangle$,$\langle sup:1\rangle$,$\langle \delta :0\rangle$,$\langle d:1,c:1\rangle \rangle$)
*c*	*fbdc* : 1	($\langle \langle fb\rangle$,$\langle sup:1\rangle$,$\langle \delta :0\rangle$,$\langle d:1,c:1\rangle \rangle$)
*c*	*gebc* : 1	($\langle \langle gbe\rangle$,$\langle sup:1\rangle$,$\langle \delta :1\rangle$,$\langle d:0,c:1\rangle \rangle$)
*d*	*fgd* : 2	($\langle \langle fg\rangle$,$\langle sup:1\rangle$,$\langle \delta :1\rangle$,$\langle d:1,c:0\rangle \rangle$)
*d*	*fbd* : 1	Ignored, as it is prefix of pattern (*fbdc* : 1). The support of *fbd* : 1 becomes zero after subtracting its support from the support of *fbdc* : 1.

**Fig. 2:**
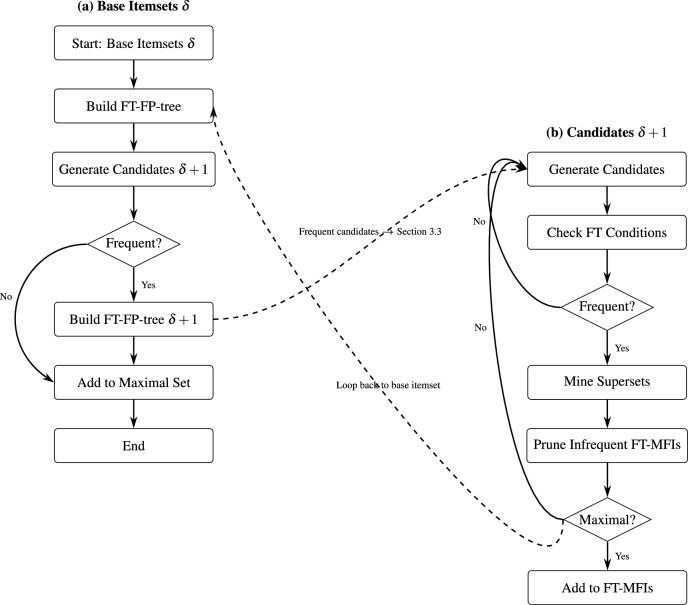
Visual workflow diagram summarizing the FT-MFI-PG algorithm. The diagram illustrates the complete process, from transaction mapping and candidate generation to the construction of FT-FP-trees, FT condition checking, superset mining, pruning heuristics, and the final maximal FT frequent itemset output. (**a**) Shows the generation of FT-FP-trees for base itemsets of length $\delta$, and (**b**) shows the recursive mining of itemsets of length $(\delta +1)$ and mining maximal FT frequent itemsets.

### Constructing fault-tolerant (FT) frequent pattern tree (FT-FP-tree)

The proposed algorithm discovers the complete set of maximal FT frequent itemsets (FT-MFIs) by creating an enhanced tree structure referred to as the FT-FP-tree (Fault-Tolerant Frequent Pattern Tree). Conceptually, the FT-FP-tree extends the traditional FP-tree ^22,49,50^ by incorporating FT conditional patterns. This enhancement allows the algorithm to efficiently prune infrequent items that do not appear in the dataset, thereby focusing computation on promising candidate itemsets.

An FT conditional pattern of an itemset *X* is defined as a conditional transaction that contains at least $(|X|-\delta )$ items of *X*, i.e., it satisfies the fault-tolerant matching constraint. Each FT conditional pattern is represented as a 4-tuple:

$$\langle \langle P\rangle ,\langle sup\rangle ,\langle \delta _p\rangle ,\langle item_1:sup,\ item_2:sup,...,item_n:sup\rangle \rangle$$

where each component has a precise meaning. The first component *P* denotes the prefix itemset (i.e., the items that can be used to generate supersets of *X*) and is mapped onto the corresponding branch of the FT-FP-tree. The second component *sup* is the support of the conditional pattern, representing the number of transactions in the database that contribute to this pattern. The third component $\delta _p$, referred to as the *row FT value*, explicitly represents the number of missing items of *X* in the current conditional pattern. Thus, $\delta _p = 0$ indicates that all items of *X* are present in the pattern, whereas $\delta _p = 1$ indicates that exactly one item of *X* is missing. This value is not the maximum tolerance threshold; rather, it is the observed fault level associated with the specific conditional pattern. The fourth component stores the *item-level support distribution* of the items in *P*. Specifically, for each individual item $item_i \in P$, the entry $\langle item_i:sup\rangle$ represents the number of transactions (captured by this conditional pattern) in which item $item_i$ occurs. For example, the vector $\langle d:1, c:1\rangle$ indicates that within this conditional pattern, both *d* and *c* contribute a support count of 1, while $\langle d:0, c:1\rangle$ indicates that *c* contributes support 1 but *d* does not occur in this conditional pattern. These values correspond to the support contributions of the current conditional pattern only. The overall item-level FT support ($item\_sup^{\delta }$) is then obtained by aggregating the contributions of $item_i$ across all FT conditional patterns in *X*. To organize these components efficiently, the leaves of the FT-FP-tree maintain FT conditional pattern tables (FT-CP-Tables). Each FT-CP-Table contains three columns corresponding to the second, third, and fourth components of the FT conditional pattern, respectively. This tabular mapping ensures that all necessary information for evaluating both itemset and individual item support is readily accessible during the mining process.

“Constructing FT-FP-tree of base itemsets of length $(\delta )$” and “Mining maximal FT frequent itemsets from FT-FP-tree” sections describe how the proposed algorithm mines the complete set of maximal FT itemsets from the FT-FP-tree. “Constructing FT-FP-tree of base itemsets of length $(\delta )$” section explains how the algorithm constructs the FT-FP-tree for itemsets of length $\delta$ by extracting and transforming conditional patterns from the FP-tree. “Mining maximal FT frequent itemsets from FT-FP-tree” section then extends this process to itemsets of length $(\delta + 1)$. In this step, the algorithm mines maximal FT frequent itemsets using information from both the FT-FP-tree of length $\delta$ and the global FP-tree. This approach ensures that all valid supersets are discovered while maintaining consistency in the FT mining process.

Before discussing the procedures in “Constructing FT-FP-tree of base itemsets of length $(\delta )$” and “Mining maximal FT frequent itemsets from FT-FP-tree” sections, it is important to explain why the algorithm constructs FT-FP-trees for itemsets of lengths $\delta$ and $(\delta + 1)$. This design is motivated by two interconnected aspects of FT frequent itemset mining: (i) determining the correct starting point for the mining process based on the FT factor $\delta$, and (ii) ensuring the complete discovery of supersets during maximal FT frequent itemset mining. In traditional exact FP-growth ^49^, the algorithm begins by constructing FP-trees for 1-item conditional patterns, which corresponds to itemsets of length $(\delta +1)$ because in exact frequent itemset mining the $\delta$ is 0. This requires scanning the global FP-tree to extract the conditional pattern bases needed for building these initial trees. This procedure is intrinsic to pattern growth, because constructing itemsets at the next level requires scanning the tree of the previous level. The proposed algorithm follows the same principle. In FT itemset mining, all itemsets whose lengths are less than or equal to $\delta$ are automatically FT-frequent. Therefore, mining must begin from itemsets of length $(\delta +1)$, which are the smallest non-trivial FT itemsets. To correctly initialize mining at this level, the algorithm scans the global FP-tree once to extract the FT conditional patterns required for constructing the FT-FP-tree of itemsets of length $(\delta +1)$. To support this initialization, the algorithm first constructs the FT-FP-tree for itemsets of length $\delta$ (“Constructing FT-FP-tree of base itemsets of length $(\delta )$” section), and then constructs the FT-FP-tree for itemsets of length $(\delta +1)$ (“Mining maximal FT frequent itemsets from FT-FP-tree” section). This process mirrors the natural progression of traditional FP-growth and does not conflict with the independence of conditional pattern bases.

It should be note here, that although the algorithm could directly construct the FT-FP-tree for itemsets of length $(\delta +1)$, the intermediate construction at level $\delta$ is significantly more efficient. First, they establish the boundary from which valid FT supersets can be generated. Second, they ensure that the FT-FP-tree for itemsets of length $(\delta + 1)$ correctly incorporates all FT conditional patterns associated with their immediate parent structures. Without first consolidating the FT conditional patterns at level $\delta$, the algorithm would face several inefficiencies: it would need to repeatedly recompute missing item counts for each $(\delta + 1)$ candidate, and it would repeatedly recompute overlapping supports. These repeated computations would increase initialization overhead and diminish the structural advantages of the FP-tree representation. For this reason, the algorithm performs an intermediate step in “Constructing FT-FP-tree of base itemsets of length $(\delta )$” section, where FT-FP-trees for itemsets of length $\delta$ are constructed once using the global FP-tree. This step does not introduce an additional loop-back; instead, it follows the same initialization logic as traditional FP-growth. In both cases, the global FP-tree must be scanned once to extract the conditional patterns, which then seed the first non-trivial level of mining. “Mining maximal FT frequent itemsets from FT-FP-tree” section then uses the consolidated FT-FP-trees of length $\delta$, together with information from the global FP-tree, to build the FT-FP-trees for itemsets of length $(\delta + 1)$. After this initialization, all FT pattern growth proceeds entirely within the FT-FP-tree hierarchy, without requiring further traversal of the global FP-tree. Algorithm 1 provides the pseudocode for constructing the FT-FP-tree for an itemset, and Fig. 2 presents a workflow diagram summarizing the overall FT-MFI-PG procedure.

### Constructing FT-FP-tree of base itemsets of length $(\delta )$

The algorithm begins by constructing FT-FP-trees for all possible $(\delta )$-item combinations using only items that satisfy the minimum support thresholds. From these initial FT-FP-trees, the algorithm extracts FT conditional patterns, which are subsequently used to generate longer candidate itemsets.

#### Example

To illustrate the construction of an FT-FP-tree for a specific itemset, consider ${X}=(dc)$ with a FT factor $\delta =2$, $min\_sup^{\delta }=3$, and $item\_sup^{\delta }=2$. The algorithm begins by extracting the conditional patterns of the individual items *c* and *d* from the initial FP-tree. These conditional patterns are then transformed into FT conditional patterns, which embed additional information about missing items and their fault levels.

- *FT conditional patterns for item* *c*: For item *c*, the algorithm identifies three conditional patterns: $\langle fgdc:1\rangle$, $\langle fbdc:1\rangle$, and $\langle gbec:1\rangle$. Each of these is then converted into an FT conditional pattern that explicitly records four components: (i) the items available for generating supersets, (ii) the overall support of the pattern, (iii) the fault-tolerant factor $\delta$, and (iv) the support distribution of individual items in *X*. For instance, the pattern $\langle fgdc:1\rangle$ is mapped to $\langle \langle fg\rangle , \langle sup:1\rangle , \langle \delta :0\rangle , \langle d:1,c:1\rangle \rangle$, where the items *ef* are available for extending (*dc*), the support count is 1, $\delta =0$ indicates that both *d* and *c* are present, and the last component confirms that each item in (*dc*) has a support of 1. Likewise, the pattern $\langle fbdc:1\rangle$ becomes $\langle \langle fb\rangle , \langle sup:1\rangle , \langle \delta :0\rangle , \langle d:1,c:1\rangle \rangle$, while $\langle gbec:1\rangle$ is transformed into $\langle \langle gbe\rangle , \langle sup:1\rangle , \langle \delta :1\rangle , \langle d:0,c:1\rangle \rangle$, where $\delta =1$ reflects the absence of item *d*.
- *FT conditional patterns for item* *d*: The algorithm then proceeds to extract conditional patterns for item *d* by scanning the FP-tree again. Two patterns are obtained: $\langle fgd:2\rangle$ and $\langle fbd:1\rangle$. To ensure that no redundant patterns are stored, each new conditional pattern of item *d* ($d_X$) is compared with the conditional patterns already generated for item *c* ($c_X$). If $d_X$ is a subset of $d_X$, its support is reduced by the support of $d_X$, and any pattern with zero support after adjustment is discarded. In this example, $\langle fgd:2\rangle$ is identified as a prefix of $\langle fgdc:1\rangle$. After subtracting the overlap, its support is reduced to 1, producing the FT conditional pattern $\langle \langle fgd\rangle , \langle sup:1\rangle , \langle \delta :1\rangle , \langle d:1,c:0\rangle \rangle$. The second pattern, $\langle fbd:1\rangle$, is removed because it overlaps with an already mapped pattern for item *c* with equivalent support.

This process ensures that the FT-FP-tree retains only those FT conditional patterns that contribute to valid FT frequent itemsets. The explicit recording of $\delta$ values within each FT conditional pattern guarantees that the algorithm consistently respects FT thresholds while extending itemsets. As a result, the FT-FP-tree for ${X}=(dc)$ provides a compact, noise-resilient representation of the search space, which is critical for efficiently mining maximal FT frequent itemsets. Table 5 presents the complete set of FT conditional patterns associated with the itemset *dc*. It is important to note that the itemset (*dc*) used in this example has a length of two. According to the fundamental property of FT frequent itemset mining, all itemsets whose lengths are less than or equal to the FT factor $\delta$ are automatically considered FT frequent. This means that (*dc*) inherently satisfies the FT conditions without requiring explicit frequency validation.

After this, the algorithm constructs the FT-FP-tree for *dc* using the extracted FT conditional patterns. This tree serves as a compact data structure that supports the recursive exploration of longer supersets containing *dc*. During the construction process, items that do not meet the $item\_sup^{\delta }$ requirement, such as item *e*, are pruned. The pruning operation is not only a memory optimization but also a computational one, since it prevents the algorithm from generating and evaluating supersets that are guaranteed to be infrequent. By carefully discarding infrequent items, the algorithm focuses its efforts on only those extensions of *dc* that have the potential to form valid maximal FT frequent itemsets.

**Fig. 3:**
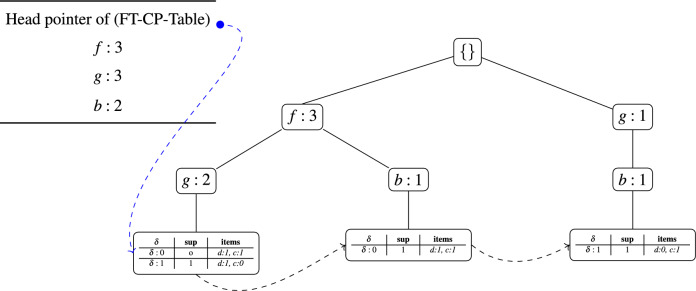
Fault-Tolerant FP-tree of itemset (*dc*).

If no supersets of *dc* are later discovered to be frequent, then *dc* itself is added to the set of FT-MFIs, ensuring that the algorithm does not overlook valid maximal patterns. This mechanism highlights the completeness property of the mining procedure: every mined FT frequent itemset is either extended into a larger frequent superset or preserved as part of the maximal set. Figure 3 illustrates the FT-FP-tree structure generated for *dc*, demonstrating how FT itemsets are organized for efficient mining. Finally, to facilitate efficient traversal and reduce repeated computation, all FT conditional pattern tables (FT-CP-Tables) associated with the FT-FP-tree are linked together to form a unified list. This linked structure allows the algorithm to navigate across related conditional patterns, enabling faster generation of supersets and efficient updating of support counts. Such design decisions are critical for scaling the algorithm to large datasets, where redundant calculations can quickly become a bottleneck.

**Algorithm 1:**
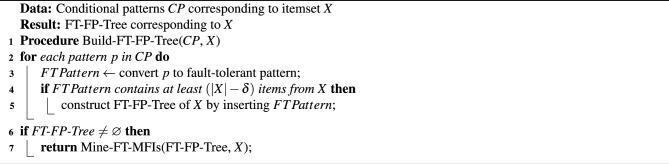
Pseudo-code for constructing FT-FP-tree for an itemset.

**Algorithm 2:**
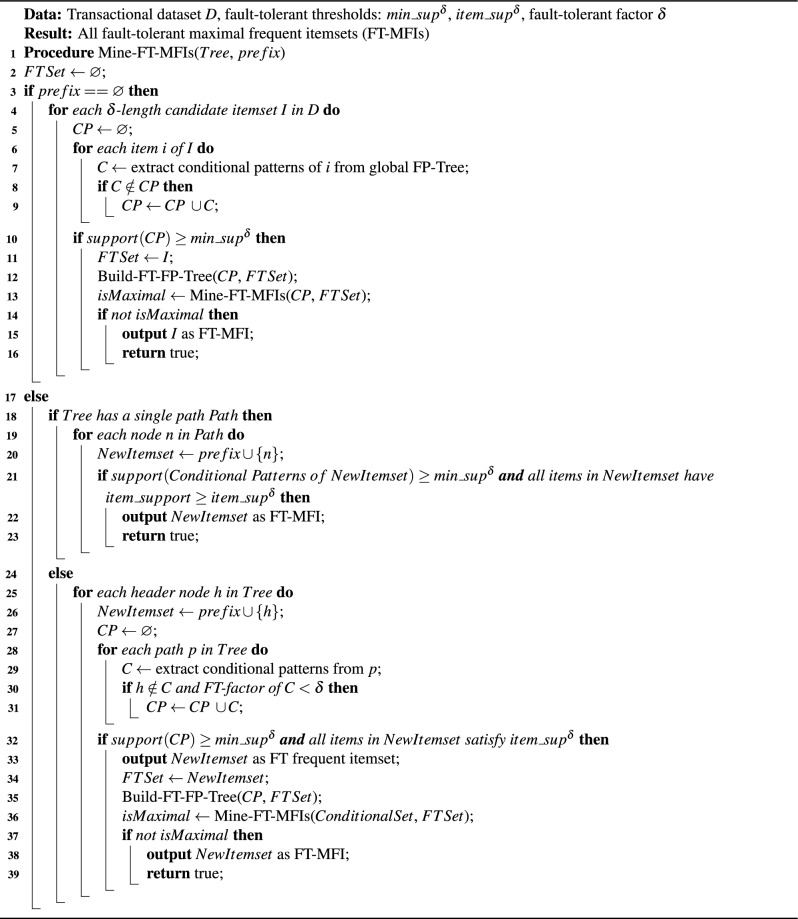
Pseudo-code for mining maximal FT frequent itemsets (FT-MFIs).

**Algorithm 3:**
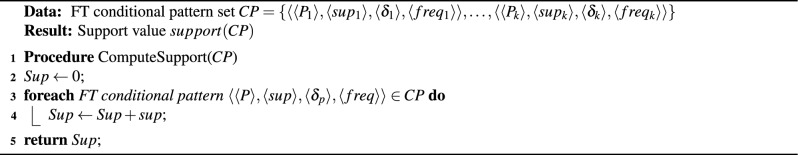
Support computation from a set of FT conditional patterns.

### Mining maximal FT frequent itemsets from FT-FP-tree

As discussed in the previous section, the FT-FP-tree constructed for an itemset *X* effectively encapsulates all transactions in which *X* appears. An important advantage of this property is that the algorithm can directly mine all supersets containing *X* from the FT-FP-tree, thereby eliminating the need to re-scan the original dataset multiple times. In practice, this significantly improves computational efficiency, especially when dealing with large or dense transactional datasets where repeated dataset scans would otherwise be prohibitively expensive. Moreover, by storing information about missing items through the FT factor $\delta$, the FT-FP-tree ensures that both exact and approximate occurrences of an itemset are considered, providing robustness against noisy or incomplete data.

#### Example

To demonstrate this process, consider the head table of the itemset (*dc*) shown in Fig. 3. From this head table, the supersets of (*dc*) can be organized based on the additional items that co-occur with it in transactions. Specifically, the candidate FT-MFIs containing (*dc*) can be divided into three distinct categories: (i) supersets that include item *b*, (ii) supersets that include item *g*, and (iii) supersets that include item *f*. These categories capture all meaningful directions in which (*dc*) can be extended within the tree structure.

The mining process then proceeds recursively. For each additional item, the algorithm extracts the corresponding conditional patterns from the FT-FP-tree and evaluates them against the FT thresholds $min\_sup^{\delta }$ and $item\_sup^{\delta }$. If the support values satisfy these thresholds, the candidate superset is retained as a valid candidate FT-MFI; otherwise, it is pruned from further exploration. For example, if (*dc*) is extended with item *b*, the algorithm checks whether (*bdc*) satisfies both global and item-level support requirements. A similar procedure applies to (*gdc*) and (*fdc*). In cases where extensions fail to meet the thresholds, those branches are discarded early, thereby reducing unnecessary computation. By traversing the FT-FP-tree in this manner, the algorithm guarantees that the complete set of FT-MFIs containing (*dc*) is discovered without performing repeated scans over the original transaction dataset. The hierarchical structure of the FT-FP-tree allows shared prefixes of transactions to be processed once and reused across multiple candidate generations, further reducing overhead.

To determine whether the itemset (*bdc*) qualifies as a maximal FT frequent itemset and to generate its possible supersets, the algorithm begins by extracting the FT conditional patterns of (*bdc*) from the FT-FP-tree of (*dc*). This step is performed by traversing all nodes of the corresponding FT-CP-Table, where each node generates a distinct FT conditional pattern for (*bdc*). Because the FT-FP-tree of (*dc*) is constructed solely from transactions containing both *c* and *d*, the extracted patterns represent only those transactions in which these two items appear together. Consequently, transactions that contain item *b* without simultaneously including *c* and *d* are absent from this structure.

To ensure completeness, the algorithm performs an additional traversal of the global FP-tree (illustrated in Fig. 1). This complementary step is essential because depending exclusively on the FT-FP-tree of (*dc*) would overlook candidate supersets of (*bdc*) that arise from transactions where *b* occurs independently of *c* and *d*. By re-examining the global FP-tree, the algorithm captures such cases and integrates them into the mining process. During this traversal, three conditional patterns for item *b* are identified: $\langle fgb:1\rangle$, $\langle fb:1\rangle$, and $\langle geb:2\rangle$. These conditional patterns ensure that the representation of (*bdc*) is now comprehensive, covering both joint occurrences of (*dc*) with *b* and occurrences where *b* appears in transactions without *c* or *d*.

A redundancy check is then applied to avoid duplicate representation of patterns already captured under other itemsets. Specifically, $\langle fb:1\rangle$ and $\langle geb:2\rangle$ are compared with conditional patterns of item *c* previously extracted from the FT-FP-tree of (*dc*) (e.g., $\langle fbdc:1\rangle$ and $\langle gebc:1\rangle$, as listed in Table 5). The pattern $\langle fb:1\rangle$ has identical support to $\langle fbdc:1\rangle$ and is therefore ignored to prevent redundancy. Similarly, the support of $\langle geb:2\rangle$ overlaps with $\langle gebc:1\rangle$, and after subtracting the overlapping support, its revised contribution is updated to 1.

**Fig. 4:**
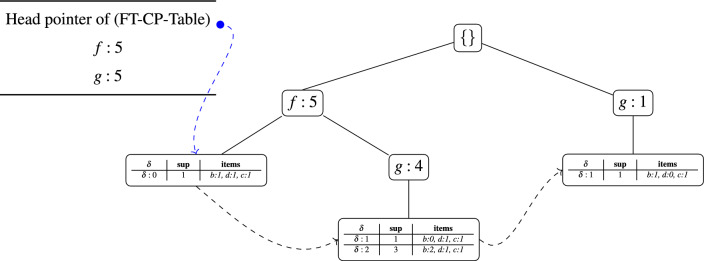
Fault-tolerant FP-tree constructed for itemset (*bdc*).

By combining the FT conditional patterns derived from both sources (the FT-CP-Table of (*dc*) and the FP-tree traversal for item (*b*)), the algorithm builds a complete and non-redundant set of conditional patterns for (*bdc*). This dual-traversal strategy ensures that no valid supersets of (*bdc*) are overlooked, while redundant or overlapping patterns are systematically pruned. The resulting FT conditional patterns form the basis for evaluating whether (*bdc*) satisfies the required thresholds ($min\_sup^{\delta }$ and $item\_sup^{\delta }$) and for mining all possible supersets of (*bdc*) in the FT-MFI framework. The extracted patterns are summarized as follows:

- $$\langle \langle ef\rangle$$,$$\langle sup:1\rangle$$,$$\langle \delta :0 \rangle$$,$$\langle d:1,c:1\rangle \rangle$$,
- $$\langle \langle fg\rangle$$,$$\langle sup:1\rangle$$,$$\langle \delta :1 \rangle$$,$$\langle d:1,c:0\rangle \rangle$$,
- $$\langle \langle fb\rangle$$,$$\langle sup:1\rangle$$,$$\langle \delta :0 \rangle$$,$$\langle d:1,c:1\rangle \rangle$$,
- $$\langle \langle geb\rangle$$,$$\langle sup:1\rangle$$,$$\langle \delta :1 \rangle$$,$$\langle d:0,c:1\rangle \rangle$$, and
- $$\langle \langle fgb\rangle$$,$$\langle sup:2\rangle$$,$$\langle \delta :2 \rangle$$,$$\langle d:0,c:0\rangle \rangle$$,

Table 6 shows the complete set of FT conditional patterns for the itemset (*bdc*). All patterns satisfy the FT factor requirement of $\delta = 2$. The itemset (*bdc*) also meets the minimum FT support threshold, $min\_sup^{\delta } = 3$, which means it appears at least three times in the transactions when considering the FT criteria. Looking at the support of individual items in the FT conditional patterns, we find that item *c* occurs in 3 transactions, item *d* occurs in 3 transactions, and item *b* occurs in 4 transactions. Since each of these counts is greater than the item-level FT support threshold, $item\_sup^{\delta } = 2$, it is clear that all items in (*bdc*) individually satisfy the required support condition. Therefore, the itemset (*bdc*) can be identified as a FT frequent itemset of length three. Figure 4 illustrates the FT-FP-tree constructed for the itemset (*bdc*), which serves as the basis for generating its supersets. To facilitate efficient traversal and mining, the algorithm links all FT-CP-Tables located at the leaf nodes of the FT-FP-tree, forming a linked list of FT-CP-Tables.

**Table 6:** Fault-tolerant conditional patterns for itemset (*bdc*).

FT conditional patterns derived from the FT-FP-Tree of itemset (*dc*)	FT conditional patterns used to construct the FT-FP-Tree of itemset (*bdc*)
$\langle \langle ef\rangle$,$\langle sup:1\rangle$,$\langle \delta :0 \rangle$,$\langle d:1,c:1\rangle \rangle$	$\langle \langle fg\rangle$,$\langle sup:1\rangle$,$\langle \delta :1 \rangle$,$\langle b:0,d:1,c:1\rangle \rangle$
$\langle \langle fg\rangle$,$\langle sup:1\rangle$,$\langle \delta :1 \rangle$,$\langle d:1,c:0\rangle \rangle$	$\langle \langle fg\rangle$,$\langle sup:1\rangle$,$\langle \delta :2 \rangle$,$\langle b:0,d:1,c:0\rangle \rangle$
$\langle \langle fb\rangle$,$\langle sup:1\rangle$,$\langle \delta :0 \rangle$,$\langle d:1,c:1\rangle \rangle$	$\langle \langle f\rangle$,$\langle sup:1\rangle$,$\langle \delta :0 \rangle$,$\langle b:1,d:1,c:1\rangle \rangle$
$\langle \langle gb\rangle$,$\langle sup:1\rangle$,$\langle \delta :1 \rangle$,$\langle d:0,c:1\rangle \rangle$	$\langle \langle g\rangle$,$\langle sup:1\rangle$,$\langle \delta :1 \rangle$,$\langle b:1,d:0,c:1\rangle \rangle$
$\langle \langle fgb\rangle$,$\langle sup:2\rangle$,$\langle \delta :2 \rangle$,$\langle d:0,c:0\rangle \rangle$	$\langle \langle fg\rangle$,$\langle sup:1\rangle$,$\langle \delta :2 \rangle$,$\langle b:2,d:0,c:0\rangle \rangle$

After identifying (*bdc*) as a FT frequent itemset, the algorithm proceeds to generate its supersets using the corresponding FT-FP-tree, as shown in Fig. 4. The candidate supersets of (*bdc*) are divided into two groups based on the additional item included: (1) supersets that contain item *g*, and (2) supersets that contain item *f*. This grouping allows the algorithm to systematically explore possible FT-MFIs.

To build and evaluate these supersets, such as (*gbdc*), the algorithm checks the FT support conditions, $min\_sup^{\delta }$ and $item\_sup^{\delta }$, by traversing all nodes of the FT-CP-Tables linked to the FT-FP-tree of (*bdc*). Conditional patterns that include only the additional item *g* without *b*, *d*, or *c* are ignored. This is because such patterns have a FT factor of $\delta = 3$, which is higher than the allowed threshold ($\delta = 2$). As a result, they do not contribute to the support of candidate itemsets of length four. The FT conditional patterns obtained from both the FT-CP-Tables of (*bdc*) and the original FP-tree are listed below.

**Fig. 5:**
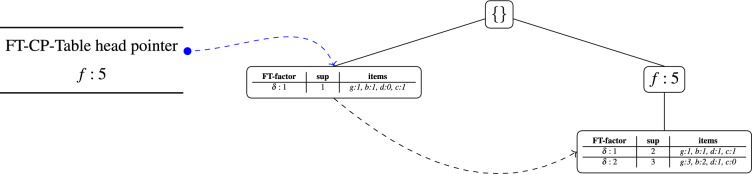
Fault-tolerant FP-tree constructed for itemset (*gbdc*).

- $$\langle \langle fg\rangle$$,$$\langle sup:1\rangle$$,$$\langle \delta :1 \rangle$$,$$\langle b:0,d:1,c:1\rangle \rangle$$,
- $$\langle \langle fg\rangle$$,$$\langle sup:3\rangle$$,$$\langle \delta :2 \rangle$$,$$\langle b:2,d:1,c:0\rangle \rangle$$,
- $$\langle \langle f\rangle$$,$$\langle sup:1\rangle$$,$$\langle \delta :0 \rangle$$,$$\langle b:1,d:1,c:1\rangle \rangle$$, and
- $$\langle \langle g\rangle$$,$$\langle sup:1\rangle$$,$$\langle \delta :1 \rangle$$,$$\langle b:1,d:0,c:1\rangle \rangle$$,

All relevant conditional patterns are converted into FT conditional patterns for the itemset (*gbdc*), and the results are shown in Table 7. Each conditional pattern satisfies the FT parameter $\delta = 2$, which ensures that the patterns allow for up to two missing items while still being considered valid. The itemset (*gbdc*) also meets the minimum FT support threshold, $min\_sup^{\delta } = 6$, meaning that it appears frequently enough in the transactions under the FT criteria. The supports of the individual items within these patterns are as follows: *g* occurs in 5 transactions, *b* in 4 transactions, *d* in 3 transactions, and *c* in 3 transactions. Since all of these values are greater than the item-level FT support requirement, $item\_sup^{\delta } = 2$, Therefore, the itemset (*gbdc*) qualifies as a FT frequent itemset of length four. Figure 5 shows the FT-FP-tree built for (*gbdc*). To improve efficiency in traversal and superset generation, the algorithm connects all FT-CP-Tables located at the leaf nodes of the tree.

**Table 7:** Fault-tolerant conditional patterns for itemset (*gbdc*).

FT conditional patterns extracted from FT-FP-Tree for (*bdc*)	FT conditional patterns for constructing FT-FP-Tree of (*gbdc*)
$\langle \langle f\rangle$,$\langle sup:1\rangle$,$\langle \delta :0 \rangle$,$\langle b:1,d:1,c:0\rangle \rangle$	$\langle \langle f\rangle$,$\langle sup:2\rangle$,$\langle \delta :1 \rangle$,$\langle g:0,b:1,d:1,c:1\rangle \rangle$
$\langle \langle fg\rangle$,$\langle sup:1\rangle$,$\langle \delta :1 \rangle$,$\langle b:0,d:1,c:1\rangle \rangle$	$\langle \langle f\rangle$,$\langle sup:1\rangle$,$\langle \delta :1 \rangle$,$\langle g:1,b:0,d:1,c:1\rangle \rangle$
$\langle \langle fg\rangle$,$\langle sup:3\rangle$,$\langle \delta :2 \rangle$,$\langle b:2,d:1,c:0\rangle \rangle$	$\langle \langle f\rangle$,$\langle sup:3\rangle$,$\langle \delta :2 \rangle$,$\langle g:3,b:2,d:1,c:0\rangle \rangle$
$\langle \langle g\rangle$,$\langle sup:1\rangle$,$\langle \delta :1 \rangle$,$\langle b:1,d:0,c:1\rangle \rangle$	$\langle \langle \rangle$,$\langle sup:1\rangle$,$\langle \delta :1 \rangle$,$\langle g:1,b:1,d:0,c:1\rangle \rangle$

**Table 8:** Fault-tolerant conditional patterns for itemset (*fgbdc*).

FT conditional patterns obtained from the FT-FP-tree of itemset (*gbdc*)	FT conditional patterns for building FT-FP-tree of (*fgbdc*)
$\langle \langle \rangle$,$\langle sup:1\rangle$,$\langle \delta :1 \rangle$,$\langle b:1,d:1,c:0\rangle \rangle$	$\langle \langle \rangle$,$\langle sup:2\rangle$,$\langle \delta :1 \rangle$,$\langle f:0,g:0,b:1,d:1,c:1\rangle \rangle$
$\langle \langle f\rangle$,$\langle sup:2\rangle$,$\langle \delta :1 \rangle$,$\langle g:1,b:1,d:1,c:1\rangle \rangle$	$\langle \langle \rangle$,$\langle sup:2\rangle$,$\langle \delta :1 \rangle$,$\langle f:2,g:1,b:1,d:1,c:1\rangle \rangle$
$\langle \langle f\rangle$,$\langle sup:3\rangle$,$\langle \delta :2 \rangle$,$\langle g:3,b:2,d:1,c:0\rangle \rangle$	$\langle \langle \rangle$,$\langle sup:3\rangle$,$\langle \delta :2 \rangle$,$\langle f:3,g:3,b:2,d:1,c:0\rangle \rangle$

Using the same procedure as with the itemset (*gbdc*), the algorithm now generates candidate supersets for (*fgbdc*). From the FT conditional patterns of (*gbdc*) (see Table 7), it is clear that item *f* is the only additional frequent item available. Therefore, the algorithm concentrates on building candidate itemsets that include *f*. To construct the FT conditional patterns for (*fgbdc*), the algorithm traverses all nodes in the linked list of FT-CP-Tables associated with (*gbdc*). Conditional patterns that include only the additional item *f* without *g*, *b*, *d*, or *c* are ignored. This is because such patterns have a FT factor of $\delta = 4$, which is higher than the allowed threshold ($\delta = 2$). As a result, they do not contribute to the support of candidate itemsets of length five. The list below presents the FT conditional patterns obtained through this process.

- $$\langle \langle e\rangle$$,$$\langle sup:2\rangle$$,$$\langle \delta :1 \rangle$$,$$\langle g:1,b:1,d:2,c:2\rangle \rangle$$,
- $$\langle \langle e\rangle$$,$$\langle sup:3\rangle$$,$$\langle \delta :2 \rangle$$,$$\langle g:3,b:2,d:1,c:0\rangle \rangle$$, and
- $$\langle \langle \rangle$$,$$\langle sup:1\rangle$$,$$\langle \delta :1 \rangle$$,$$\langle g:1,b:1,d:0,c:1\rangle \rangle$$,

All identified patterns are transformed into FT conditional patterns for the itemset (*fgbdc*), as summarized in Table 8. Each conditional pattern adheres to the FT factor ($\delta =2$), which establishes a support threshold of six for the itemset. Thus, the itemset (*fgbdc*) satisfies the required $min\_sup^{\delta }$ threshold. Table 8 presents the complete set of FT conditional patterns for (*fgbdc*). The support counts for individual items, derived from these patterns, are: $\langle f:5\rangle$, $\langle g:5\rangle$, $\langle b:4\rangle$, $\langle d:3\rangle$, and $\langle c:3\rangle$. Each of these items meets or exceeds the item-level support threshold ($item_sup^{\delta }=2$). Consequently, (*fgbdc*) is validated as a FT frequent itemset of length five.

As no supersets of (*fgbdc*) exist, the algorithm classifies it as a maximal FT frequent itemset and adds it to the set of FT-MFIs. Because (*fgbdc*) is a superset of previously identified FT frequent itemsets, including (*gbdc*), (*bdc*), and (*dc*), these subsets are pruned from the FT-MFIs set to preserve maximality. Following this step, the algorithm backtracks to process the next $\delta$-length itemset (*ec*). Mirroring the procedure demonstrated for itemset (*dc*) in the prior example, the algorithm recursively constructs FT-FP-trees for the remaining itemsets and mines them to discover all remaining FT-MFIs. Algorithm 2 shows the pseudo code for extracting the entire set of maximal FT frequent itemsets.

### Time and space complexity

The search space for mining maximal FT-frequent itemsets can grow up to $2^{m}$ possible itemsets for $m$ distinct items, making the problem inherently exponential in the worst case. In the proposed algorithm, itemsets are expanded recursively using the FT-FP-tree, and their FT-support is computed directly from the FT-FP-tree together with the corresponding FT conditional patterns (projected databases). Consequently, the total running time depends on the amount of work spent across all explored itemsets, which can be expressed as

$$O\!\left( \sum _{I \in {S}} |D_{I}|\right)$$

where $|D_{I}|$ denotes the size of the projected database for itemset $I$, and ${S}$ is the set of prefix itemsets that survive HUT-based pruning.

In dense datasets, many item combinations remain frequent, and pruning becomes less effective. As a result, the algorithm may need to explore a large number of itemsets, and the complexity can approach the exponential bound $O(N \cdot 2^{m})$, where $N$ is the number of transactions. In typical datasets, however, the FT-FP-tree structure, shared FT conditional patterns, and strong upper-bound pruning significantly reduce the number of itemsets that must be explored, making the pattern-growth process far more efficient in practice. The space complexity is dominated by the FT-FP-tree and the FT conditional patterns generated during recursive processing. In the worst case, when no prefix compression is possible, the FT-FP-tree may need to store every item occurrence, which leads to a space requirement of $O(N \cdot d)$, where $d$ is the average transaction length. Because projected databases are stored in a compact form and reused across recursive calls, the additional working memory beyond the input and output is generally limited to $O(N + m)$, which includes the header table, temporary structures, and the recursion stack.

## Correctness and completeness of FT-MFI-PG

In this section, we provide a rigorous proof framework to justify the correctness of the proposed FT-MFI-PG algorithm. Specifically, we formally justify: (i) the boundary condition that itemsets with length $\le \delta$ are automatically FT-frequent, (ii) the completeness of the recursive FT mining procedure (i.e., no valid FT-MFI is missed), and (iii) the maximal correctness of the output set (i.e., every reported pattern is maximal).

### Preliminaries and definitions

Let *D* be a transaction database and let *X* be an itemset. According to the FT itemset mining model ^35^, a transaction $T \in D$ is said to be an FT transaction of *X* if

1 $$\begin{aligned} |T \cap X| \ge |X| - \delta \end{aligned}$$

The FT support of *X* is defined as the number of FT transactions of *X* in *D*:

2 $$\begin{aligned} sup^{\delta }(X) = |\{T \in D \mid |T \cap X| \ge |X| - \delta \}| \end{aligned}$$

An itemset *X* is an FT frequent itemset if

3 $$\begin{aligned} sup^{\delta }(X) \ge (min\_sup^{\delta }) \end{aligned}$$

and additionally, each item $i \in X$ must occur in at least $(item\_sup^{\delta })$ FT transactions of *X*, i.e.,

4 $$\begin{aligned} sup^{\delta }(i,X) \ge (item\_sup^{\delta }), \;\;\; \forall i \in X \end{aligned}$$

where $sup^{\delta }(i,X)$ denotes the number of FT transactions of *X* that contain item *i*. Finally, an FT frequent itemset *X* is called a maximal FT frequent itemset (FT-MFI) if there exists no FT frequent itemset *Y* such that

5 $$\begin{aligned} X \subset Y \end{aligned}$$

### Boundary condition: why $|X| \le \delta$ is automatically FT-frequent

#### Lemma 1

(Automatic FT Transaction Condition for Small Itemsets) *Let* *X* *be an itemset such that* $|X| \le \delta$. *Then for every transaction* $T \in D$, *T* *is an FT transaction of* *X*.

#### Proof

From Eq. 1, *T* is an FT transaction of *X* if

$$|T \cap X| \ge |X| - \delta$$

Since $|X| \le \delta$, we have $|X|-\delta \le 0$. For any transaction *T*, it always holds that $|T \cap X| \ge 0$. Thus,

$$|T \cap X| \ge 0 \ge |X|-\delta$$

which implies that every transaction $T \in D$ satisfies the FT condition of *X*. $\square$

#### Corollary 4.1

(FT Support for Itemsets of Length $\le \delta$) *If* $|X| \le \delta$, *then*

$$sup^{\delta }(X) = |D|$$

#### Proof

By Lemma 1, all transactions in *D* satisfy the FT condition for *X*. Hence, the FT support of *X* equals the total number of transactions in *D*. $\square$

#### Corollary 4.2

(Boundary Condition for FT-Frequency) *If* $|X| \le \delta$ *and* $(min\_sup^{\delta }) \le |D|$, *then* *X* *is an FT frequent itemset*.

#### Proof

From Corollary 4.1, $sup^{\delta }(X)=|D|$. Since $(min\_sup^{\delta }) \le |D|$, we obtain

$$sup^{\delta }(X) = |D| \ge (min\_sup^{\delta }).$$

Thus, *X* satisfies the FT frequency condition. $\square$

#### Remark 4.3

*(Clarification of Boundary Conditions)* Corollary 4.2 clarifies the boundary condition used in FT-MFI-PG. All itemsets of length $\le \delta$ are automatically FT-frequent holds under the practical assumption that $(min\_sup^{\delta }) \le |D|$. If $(min\_sup^{\delta }) > |D|$, then no itemset can satisfy the FT support threshold, including those with $|X| \le \delta$.

#### Lemma 2

(Smallest Non-Trivial FT Frequent Itemsets) *The smallest non-trivial FT frequent itemsets that require explicit mining have length* $|X|=\delta +1$.

#### Proof

For $|X|=\delta +1$, Eq. 1 becomes

$$|T \cap X| \ge |X|-\delta = (\delta +1)-\delta = 1$$

Thus, only transactions containing at least one item of *X* contribute to its FT support, and the FT support of *X* depends on the actual distribution of items in *D*. Therefore, explicit mining is required. In contrast, for $|X| \le \delta$, Lemma 1 shows that every transaction supports *X* automatically. $\square$

### Completeness of the recursive mining procedure

#### Lemma 3

(Completeness of FT Conditional Pattern Generation) *Let* *X* *be an FT frequent itemset and let* *W* *be an item such that* $X \cup \{W\}$ *is also FT frequent. Then all FT conditional patterns required to construct* $X \cup \{W\}$ *are contained in the conditional pattern base of* *W*.

#### Proof

If $X \cup \{W\}$ is FT frequent, then it is supported by at least $(min\_sup^{\delta })$ FT transactions, i.e.,

$$sup^{\delta }(X \cup \{W\}) \ge (min\_sup^{\delta })$$

Thus, there exist at least $(min\_sup^{\delta })$ transactions $T \in D$ satisfying

$$|T \cap (X \cup \{W\})| \ge |X \cup \{W\}| - \delta$$

For each such transaction, the items co-occurring with *W* form part of the prefix path of *W* in the global FP-tree. Therefore, every FT occurrence contributing to the FT support of $X \cup \{W\}$ is captured in the conditional pattern base of *W*. Hence, the conditional pattern base contains all necessary information to generate FT extensions including $X \cup \{W\}$. $hfill\square$

#### Lemma 4

(No Missing FT Frequent Itemsets) *If an itemset* *X* *is an FT frequent itemset, then FT-MFI-PG will generate and evaluate* *X* *during the recursive mining process*.

#### Proof

FT-MFI-PG follows a pattern-growth strategy similar to FP-growth ^49^. Mining starts from itemsets of size $\delta +1$ (Lemma 2) and recursively grows patterns using conditional pattern bases. At each recursion step, the FT conditional pattern base contains all transactions contributing to the FT support of the current prefix (Lemma 3). Therefore, any FT frequent itemset *X* can be constructed through successive extensions and will be evaluated during the recursion. Hence, no FT frequent itemset is missed. $\square$

#### Theorem 4.4

(Completeness of FT-MFI-PG) *FT-MFI-PG enumerates all maximal FT frequent itemsets in the database* *D*.

#### Proof

Let *M* be any maximal FT frequent itemset in *D*. Since *M* is FT frequent, Lemma 4 ensures that FT-MFI-PG generates and evaluates *M*. Therefore, every maximal FT frequent itemset in *D* is discovered. Hence, FT-MFI-PG is complete. $\square$

### Maximal correctness of the output set

#### Lemma 5

(Soundness of Reported Patterns) *Every itemset reported by FT-MFI-PG satisfies both* $(min\_sup^{\delta })$ *and* $(item\_sup^{\delta })$ *constraints*.

#### Proof

FT-MFI-PG reports an itemset *X* only after computing its FT support and verifying that

$$sup^{\delta }(X) \ge (min\_sup^{\delta })$$

and

$$sup^{\delta }(i,X) \ge (item\_sup^{\delta }), \;\;\; \forall i \in X$$

Thus, every reported itemset satisfies the FT frequent itemset definition. $\square$

#### Lemma 6

(Correctness of Maximality Condition) *If FT-MFI-PG outputs an itemset* *X* *as maximal, then there exists no FT frequent itemset* *Z* *such that* $X \subset Z$.

#### Proof

Assume there exists an FT frequent itemset *Z* such that $X \subset Z$. Then there exists at least one item $i \in Z \setminus X$ such that $X \cup \{i\}$ is FT frequent. By Lemma 3, the conditional pattern base of *X* contains all FT occurrences needed to generate such an extension. Therefore, FT-MFI-PG would extend *X* during recursion and would not terminate at *X*. This contradicts the assumption that *X* is output as maximal. Hence, no FT frequent superset exists. $\square$

#### Theorem 4.5

(Maximal Correctness of FT-MFI-PG) *Every itemset reported by FT-MFI-PG is a maximal FT frequent itemset, and no non-maximal FT frequent itemset is included in the output set*.

#### Proof

From Lemma 5, each reported itemset is FT frequent. From Lemma 6, each reported itemset has no FT frequent superset. Therefore, each reported itemset is maximal by definition. Hence, the output set contains only maximal FT frequent itemsets. $\square$

### Correctness and completeness of FT-MFI-PG

#### Theorem 4.6

(Correctness and Completeness of FT-MFI-PG) *FT-MFI-PG outputs the complete set of maximal FT frequent itemsets in* *D*, *without missing any valid FT-MFI and without producing any non-maximal patterns*.

#### Proof

Theorem 4.4 proves that FT-MFI-PG discovers all maximal FT frequent itemsets (completeness). Theorem 4.5 proves that every reported itemset is maximal (maximal correctness). Therefore, FT-MFI-PG is both complete and correct. $\square$

## Pruning infrequent FT-MFIs

The FT-MFIs mining approach presented in Section 3 generates exactly the same set of candidate itemsets as the algorithm would when mining all FT frequent itemsets. However, the FT-MFIs set is typically an order of magnitude smaller than the complete set of FT frequent itemsets. This reduction enables the use of efficient heuristics to prune infrequent search spaces. In the literature on maximal frequent itemset mining, several well–known algorithms such as LCM ^26^, GenMax ^62^, MAFIA ^27^, and FPMax ^63^ exploit different pruning mechanisms under the assumption of exact support anti-monotonicity. These approaches typically rely on pruning schemes such as diffsets, closure-based pruning, PEP (Parent Equivalence Pruning), FHUT, and head–tail maximality checking (HUTMFI) to eliminate non-promising branches early during the mining process (see Table 9 for a comparative overview). Such strategies are extremely effective in exact maximal itemset mining because support monotonically decreases when an itemset is extended. However, these pruning strategies cannot be directly adopted for maximal FT frequent itemset mining. Under exact matching, the support of an itemset always decreases or remains same when the itemset is extended. This is not valid in the fault-tolerant context because an itemset $(X \cup i)$ may satisfy the FT support threshold even if *X* does not. Therefore, pruning strategies that rely on exact support, such as diffset-based pruning, closure checking, or PEP, cannot be directly transferred to the FT mining context without compromising correctness or completeness. For example, the diffset optimization used in GenMax depends strongly on the monotonic decreasing property of supports, while closure-based pruning in LCM assumes that closedness can be maintained by exact support comparisons. These conditions are violated when support is computed under $\delta$ relaxed semantics.

Despite these challenges, the FT–MFI–PG algorithm adopts pruning strategies that remain valid in the fault–tolerant setting. Specifically, FT–MFI–PG incorporates FHUT and HUTMFI heuristics, which are conceptually related to pruning schemes used in MAFIA ^27^ and GenMax ^63^, but are redesigned such that they do not require the exact anti-monotonicity of support. In addition, the FT–FP–tree combined with FT conditional pattern extraction performs prefix-based pruning and early elimination of infeasible conditional branches while preserving the $\delta$ relaxed support conditions. These components enable aggressive pruning comparable to classical maximal itemset mining algorithms, but without violating the semantics of fault tolerance.

**Table 9:** Comparison of pruning techniques used in maximal frequent itemset mining algorithms and applicability to FT-MFI. PEP is Parent Equivalence Pruning, FHUT is Frequent Head Union Tail, and HUTMFI is Head Union Tail Maximal Frequent Itemset.

Algorithm/pruning techniques	Diffsets	Closure-based pruning	PEP	FHUT	HUTMFI	Prefix-based pruning
LCM ^26^	$\times$	$\checkmark$	$\times$	$\times$	$\times$	$\checkmark$
GenMax ^62^	$\checkmark$	$\times$	$\checkmark$	$\checkmark$	$\checkmark$	$\checkmark$
MAFIA ^27^	$\times$	$\times$	$\checkmark$	$\checkmark$	$\checkmark$	$\checkmark$
FPMax ^63^	$\times$	$\times$	$\times$	$\checkmark$	$\checkmark$	$\checkmark$
FT–MFI–PG	$\times$	$\times$	$\times$	$\checkmark$	$\checkmark$	$\checkmark$

### FHUT (frequent head union tail)

 ^64^ and ^27^ observed that the longest itemset that can be mined in an iteration is the current frequent itemset combined with all frequent items in the header table. Let *H* denotes the frequent itemset of the current iteration, and *T* represents the set of all frequent items in the header table. If the union ${H} \cup {T}$ itself forms an FT-MFI, then all frequent items in the header table can be pruned from further consideration.

### HUTMFI (head union tail maximal FT frequent itemset)

Before generating conditional patterns for an itemset, the algorithm checks whether the union of *H* with the frequent items in *T* is a subset of any known FT-MFI. If it is, generation of conditional patterns for the remaining frequent items in the header table is halted, and the algorithm backtracks to the parent itemset ^27^. Experimental results indicate that applying this heuristic can yield substantial performance gains.

### Maximal superset checking

To determine whether an itemset *X* is maximal, the algorithm checks if *X* is a subset of any known FT-MFI, a process referred to as maximal superset checking. Naively, this requires comparing *X* against all known FT-MFIs, which can be computationally expensive for low support thresholds where the number of FT-MFIs is large. To improve efficiency, we adopt a local MFI (LMFI) superset checking strategy ^65^.

LMFI examines supersets using a divide-and-conquer approach. Instead of comparing *X* with all known FT-MFIs, it considers only those FT-MFIs where the parent of *X* appears as a prefix. All FT-MFIs are stored in a compact tree structure called the Max-FT-tree, which allows the algorithm to avoid exhaustive comparisons. Similar to the FP-tree, the Max-FT-tree maps FT-MFIs along the branches of the tree ^63^. Shared prefixes among multiple FT-MFIs are merged into common branches, thereby improving the efficiency of superset comparisons. Nodes with the same items are linked via linked lists, and the head pointers and linked lists are used to retrieve the mined FT-MFIs. To find supersets of *X*, the parent itemset of *X* projects a list of pointers to only those FT-MFIs in the Max-FT-tree where the parent itemset appears as a subset.

## Experiments

To assess the effectiveness and efficiency of proposed algorithm, *FT-MFI-PG*, we conducted a series of experiments comparing its performance with the most relevant existing FT-MFI mining algorithms. Specifically, we evaluated the algorithm on one synthetic dataset, *T10I4D100K*, and three widely used real-world benchmark datasets: *FoodMart*, *FoodMart*, and *BMSWebView1*. These datasets are standard choices in the frequent itemset mining (FIM) community for evaluating algorithm performance due to their varying sizes, item distributions, and transaction characteristics. All datasets are publicly accessible through the FIM repository (https://fimi.uantwerpen.be/data/ ). Table 10 presents a concise overview of these datasets, including the number of transactions, average transaction length, and the total number of distinct items in each dataset.

For benchmarking purposes, we compared *FT-MFI-PG* against two prominent FT-MFI mining algorithms: *VB-FT-Mine* ^48^ and *FT-MFI-MultiTree* ^51^. *VB-FT-Mine* is an Apriori-inspired method that traverses the search space in a top-down manner. It begins by examining individual items, gradually building up to larger itemsets while verifying the FT conditions for each candidate. To accelerate support counting, it employs bitwise operations on itemset bit-vectors ^27^. Despite its computational optimizations, *VB-FT-Mine* faces scalability challenges for large datasets because it generates an exponentially growing number of candidate itemsets and repeatedly scans the entire dataset to calculate support counts. In contrast, *FT-MFI-MultiTree* is a tree-based approach designed to handle FT by mapping transactions with missing items across multiple FP-trees ^51^. Its inefficiency mainly arises when similar transactions differ in missing items, causing repeated computations of support and increased memory usage due to redundant storage in multiple trees. Since neither *VB-FT-Mine* nor *FT-MFI-MultiTree* has publicly available implementations, we re-implemented all algorithms in C++ to ensure a fair and consistent comparison.

All experiments were carried out on a machine equipped with an Intel processor running at 3.2 GHz and 8 GB of RAM. To examine performance trends, we varied the minimum support thresholds and tested FT factors of $\delta = 1$ and $\delta = 2$. The specific parameter settings adopted for each dataset in these experiments are summarized in Table 11. This setup allowed us to evaluate the computational efficiency and scalability of *FT-MFI-PG* in comparison with the baseline FT-MFI algorithms across different dataset characteristics and parameter configurations. Since the proposed *FT-MFI-PG* algorithm is fully deterministic, running it multiple times on the same dataset with the same parameters always produces exactly the same results (number of maximal FT frequent itemsets) and the same execution time. Because there is no difference between runs, statistical measures such as standard deviation, variance, or confidence intervals are not needed and are not included.

**Table 10:** Overview of the datasets: transactions, items, and average transaction length.

Dataset	Number of transactions	Number of items	Avg. transaction length
Retail	88, 162	16, 470	10
BMSWebView1	59, 601	497	3
T10I4D100K	100, 000	870	11
FoodMart	4, 141	1, 559	4

**Table 11:** Dataset-specific fault-tolerant factors and support thresholds applied in the experiments.

Dataset	Number of transactions	$\delta$	$item\_sup^{\delta }$	$min\_sup^{\delta }$
Retail	88, 162	$1\ and\ 2$	$0.05\%\ to\ 0.30\%$	$0.35\%\ to\ 0.70\%$
BMSWebView1	59, 601	$1\ and\ 2$	$0.01\%\ to\ 0.16\%$	$0.20\%\ to\ 0.60\%$
T10I4D100K	100, 000	$1\ and\ 2$	$0.5\%\ to\ 1.5\%$	$1.7\%\ to\ 5.0\%$
FoodMart	4, 141	$1\ and\ 2$	$0.02\%\ to\ 0.06\%$	$0.09\%\ to\ 0.20\%$

**Fig. 6:**
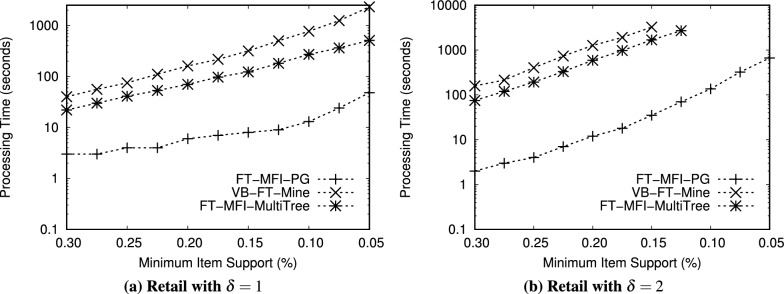
Performance comparison of FT-MFI mining algorithms on the *Retail* dataset ($min\_sup^{\delta }=0.35\%$).

**Fig. 7:**
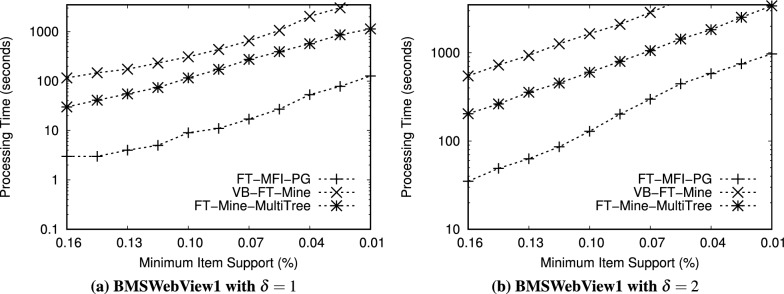
Performance comparison of FT-MFI mining algorithms on the *BMSWebView1* dataset ($min\_sup^{\delta }=0.20\%$).

**Fig. 8:**
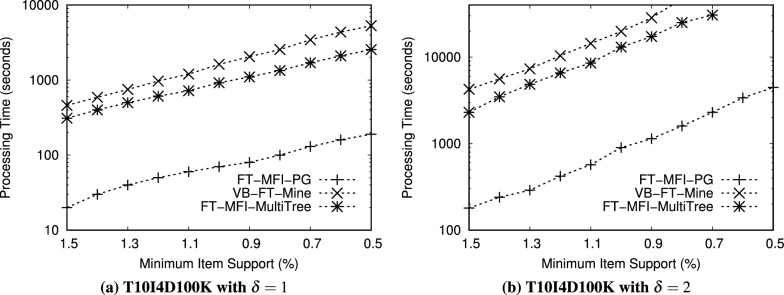
Performance comparison of FT-MFI mining algorithms on the *T10I4D100K* dataset ($min\_sup^{\delta }=1.7\%$).

**Fig. 9:**
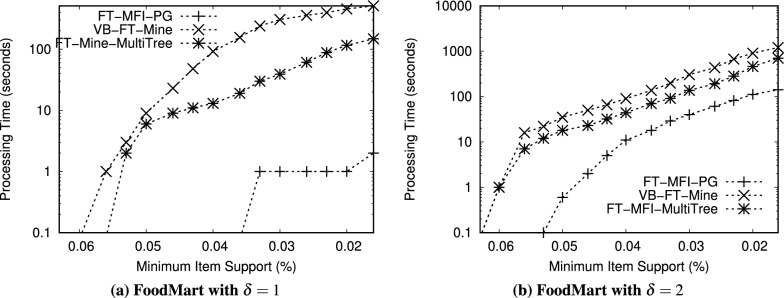
Performance comparison of FT-MFI mining algorithms on the *FoodMart* dataset ($min\_sup^{\delta }=0.09\%$).

We evaluated the performance of the FT-MFI mining algorithms through three key experiments:

- *Processing time:* The first experiment measures the total time required by each algorithm to extract the complete set of FT-MFIs, thereby reflecting their computational efficiency.
- *Scalability:* The second experiment examines how the algorithms perform on datasets of varying sizes to assess their ability to handle increasing volumes of transactional data.
- *Transaction complexity:* The third experiment analyzes execution time on subsets of datasets with different transaction lengths, highlighting the impact of transaction length on algorithmic performance.
- *Memory consumption:* The fourth experiment evaluates the amount of memory utilized by each algorithm during execution.

**Table 12:** Performance evaluation of FT-MFI mining algorithms with varying min_sup$^{\delta }$ on the *Retail* dataset and item_sup$^{\delta }$ = 0.10%. The values in the table cells represent the processing time (in seconds) required by each algorithm to mine the maximal fault-tolerant frequent itemsets.

$\delta$	min sup$^{\delta }$	FT-MFI-PG	VB-FT-Mine	FT-MFI-MultiTree
$\delta = 1$	0.35%	8	168	124
	0.40%	7	156	113
	0.50%	6	143	102
	0.60%	4	113	84
	0.70%	3	87	77
$\delta = 2$	0.35%	62	> 3,200	> 3,200
	0.40%	56	> 3,200	> 3,200
	0.50%	48	> 3,200	> 3,200
	0.60%	22	> 3,200	2988
	0.70%	12	3,123	2578

**Table 13:** Performance evaluation of FT-MFI mining algorithms with varying min_sup$^{\delta }$ on the *BMSWebView1* dataset and item_sup$^{\delta }$ = 0.04%. The values in the table cells represent the processing time (in seconds) required by each algorithm to mine the maximal fault-tolerant frequent itemsets.

$\delta$	min sup$^{\delta }$	FT-MFI-PG	VB-FT-Mine	FT-MFI-MultiTree
$\delta = 1$	0.20%	52	2145	742
	0.30%	48	1975	691
	0.40%	43	1768	643
	0.50%	35	1454	550
	0.60%	21	1139	405
$\delta = 2$	0.20%	604	>3200	1256
	0.30%	553	>3200	1199
	0.40%	498	>3200	1132
	0.50%	414	>3200	1013
	0.60%	356	3134	958

**Table 14:** Performance evaluation of FT-MFI mining algorithms with varying min_sup$^{\delta }$ on the *T10I4D100K* dataset and item_sup$^{\delta }$ = 0.70%. The values in the table cells represent the processing time (in seconds) required by each algorithm to mine the maximal fault-tolerant frequent itemsets.

$\delta$	min sup$^{\delta }$	FT-MFI-PG	VB-FT-Mine	FT-MFI-MultiTree
$\delta = 1$	1.7%	109	3145	1251
	2.0%	106	3085	1214
	3.0%	101	3047	1162
	4.0%	94	2981	1079
	5.0%	85	2893	987
$\delta = 2$	1.7%	2370	>3200	>3200
	2.0%	2325	>3200	>3200
	3.0%	2863	>3200	>3200
	4.0%	2786	>3200	>3200
	5.0%	2668	>3200	>3200

**Table 15:** Performance evaluation of FT-MFI mining algorithms with varying min_sup$^{\delta }$ on the *FoodMart* dataset and item_sup$^{\delta }$ = 0.03%. The values in the table cells represent the processing time (in seconds) required by each algorithm to mine the maximal fault-tolerant frequent itemsets.

$\delta$	min sup$^{\delta }$	FT-MFI-PG	VB-FT-Mine	FT-MFI-MultiTree
$\delta = 1$	0.09%	1	389	68
	0.12%	1	332	62
	0.15%	1	268	53
	0.18%	1	138	26
	0.20%	1	65	15
$\delta = 2$	0.09%	37	139	104
	0.12%	32	135	943
	0.15%	24	127	861
	0.18%	13	69	458
	0.20%	6	38	235

Experiments related to scalability (second experiment) and transaction complexity (third experiment) are carried out exclusively on the *Retail* and *T10I4D100K* datasets because of their sparsity and diverse transaction lengths. All algorithms are executed on these subsets using identical FT support thresholds to ensure a fair comparison. To study scalability, dataset subsets containing between 10, 000 and 90, 000 transactions are generated. For transaction complexity, the *T10I4D100K* and *Retail* datasets are divided into five subsets each, with 30,000 transactions randomly selected from the original dataset for each subset. The subsets are organized based on transaction lengths: the first subset includes transactions of length 1 to 10, the second subset covers transactions of length 11 to 20, and the remaining three subsets include transactions of length 21 to 30, 31 to 40, and 41 to 50, respectively. These subsets allow the evaluation of the algorithms on datasets of varying sizes and on transactions of differing complexity.

**Fig. 10:**
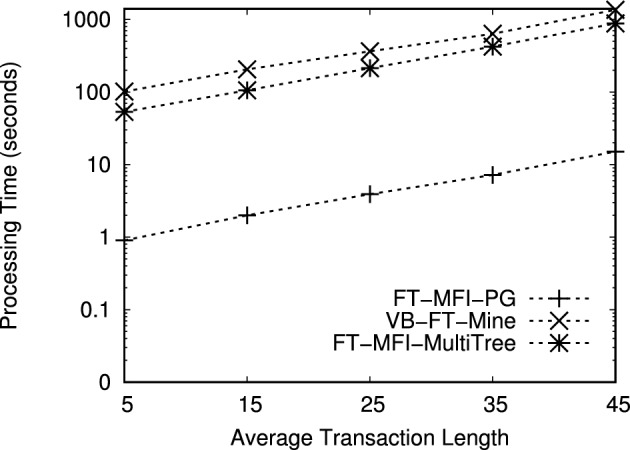
Scalability analysis of FT-MFI mining algorithms on the *Retail* dataset with varying transaction lengths ($\delta =1$, $min\_sup^{\delta }=0.05\%$, $item\_sup^{\delta }=0.35\%$).

**Fig. 11:**
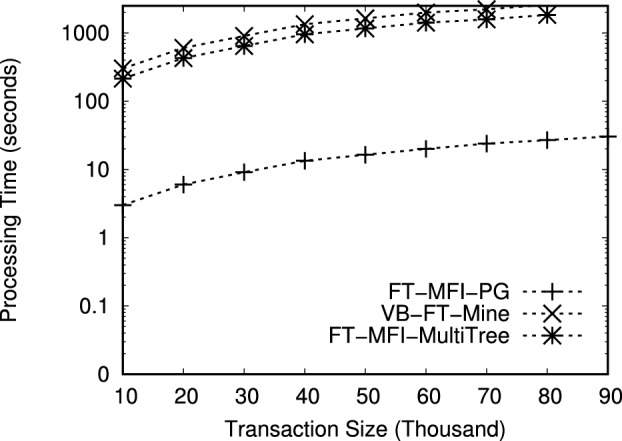
Scalability analysis of FT-MFI mining algorithms on the *Retail* dataset with varying transaction sizes ($\delta =1$, $min\_sup^{\delta }=0.05\%$, $item\_sup^{\delta }=0.35\%$).

**Fig. 12:**
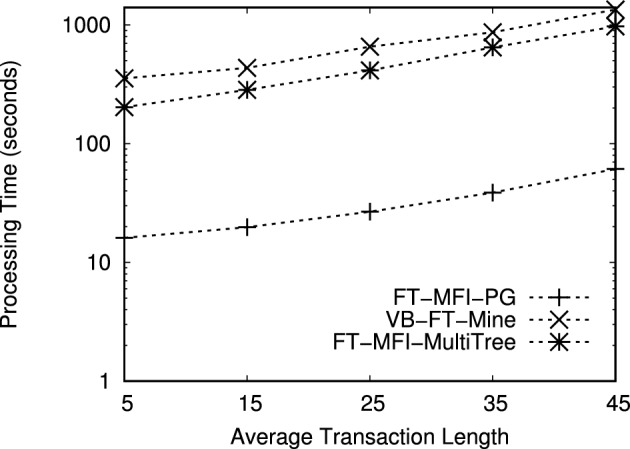
Scalability analysis of FT-MFI mining algorithms on the *T10I4D100K* dataset with varying transaction lengths ($\delta =1$, $min\_sup^{\delta }=1.4\%$, $item\_sup^{\delta }=1.7\%$).

**Fig. 13:**
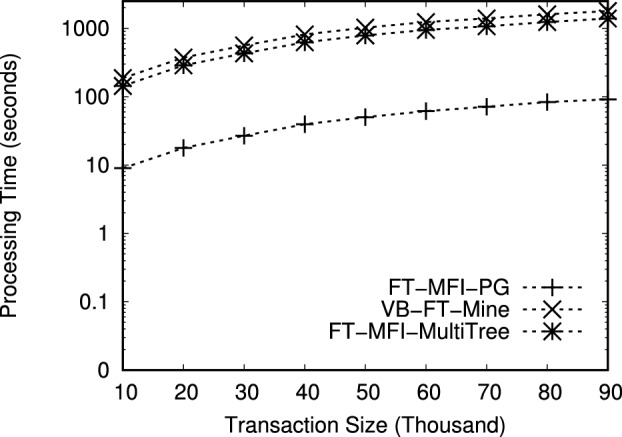
Scalability analysis of FT-MFI mining algorithms on the *T10I4D100K* dataset with varying transaction sizes ($\delta =1$, $min\_sup^{\delta }=1.4\%$, $item\_sup^{\delta }=1.7\%$).

Figures 6, 7, 8, 9 show the processing times observed for the algorithms. Here, processing time is defined as the total execution duration from the beginning of mining until the full set of FT-MFIs is obtained. As expected, all algorithms exhibit increased execution times at lower support thresholds. To maintain practicality, any experiment exceeding 3,200 seconds is terminated. Specifically, Figs. 6, 7, 8, 9 report processing times for the *Retail*, *BMSWebView1*, *FoodMart*, and *T10I4D100K* datasets. Additionally, Figs. 11 and 13 depict performance variations with respect to different dataset sizes, while Figs. 10 and 12 show the results for subsets with differing average transaction lengths.

**Systematic analysis of FT parameters** ($\delta$, min_sup$^{\delta }$, and item_sup$^{\delta }$): In the FT frequent itemset mining model, the parameter $\delta$ determines the tolerance level by allowing up to $\delta$ missing items in a transaction when evaluating whether it supports an itemset. Therefore, increasing $\delta$ relaxes the matching constraint and results in a larger number of transactions contributing to the FT support of candidate itemsets. Consequently, the search space expands rapidly, leading to a substantial increase in both the number of generated FT candidates and the computational cost of FT support verification. In addition, min_sup$^{\delta }$ and item_sup$^{\delta }$ act as frequency thresholds that control pruning strength. Since these parameters directly affect the number of frequent items retained and the number of conditional patterns constructed, they significantly influence both the output size and the processing time. To systematically evaluate the impact of these parameters, additional experiments are conducted by varying $\delta$ and min_sup$^{\delta }$ while fixing item_sup$^{\delta }$ for each dataset. The results are summarized in Tables 12, 13, 14, 15. All values in these tables represent processing time in seconds required to mine the complete set of FT-MFIs.

*Effect of* $\delta$: The results presented in figures and tables clearly indicate that increasing $\delta$ substantially increases runtime for all evaluated algorithms. This behavior is consistent with the trends observed in Figs. 6, 7, 8, 9, where the runtime increases significantly when mining is performed under relaxed FT constraints. Specifically, when $\delta$ increases from 1 to 2, more partially matching transactions satisfy the FT condition, which increases the number of FT frequent itemsets and enlarges the candidate space. For example, in the *Retail* dataset (Table 12), FT-MFI-PG requires only 3–8 seconds when $\delta =1$, but increases to 12–62 seconds when $\delta =2$. A similar trend is observed for *BMSWebView1* (Table 13), where FT-MFI-PG increases from 21–52 seconds ($\delta =1$) to 356–604 seconds ($\delta =2$). This demonstrates that $\delta$ is a dominant parameter influencing computational complexity. The impact of $\delta$ is even more severe for *VB-FT-Mine* and *FT-MFI-MultiTree*. In multiple datasets, both algorithms fail to complete within the 3,200 seconds time limit when $\delta =2$, as also reflected in Figs. 6, 7, 8, 9. This occurs because the relaxed tolerance level increases the number of candidates that must be generated and tested, causing a combinatorial explosion in Apriori-based approaches.

**Effect of min**_sup$^{\delta }$: The minimum FT support threshold min_sup$^{\delta }$ plays a critical role in controlling the number of FT frequent itemsets. Lower min_sup$^{\delta }$ values allow more itemsets to satisfy the FT frequency condition, which results in a larger output size and higher computational overhead. This observation is consistent with Figs. 6, 7, 8, 9, where all algorithms show increased runtime as support thresholds decrease. For example, in the *Retail* dataset with $\delta =2$ (Table 12), FT-MFI-PG requires 12 seconds at min_sup$^{\delta }=0.70\%$, but increases to 62 seconds at min_sup$^{\delta }=0.35\%$. Similarly, for *FoodMart* (Table 15), FT-MFI-PG increases from 6 seconds to 37 seconds when min_sup$^{\delta }$ decreases from 0.20% to 0.09% under $\delta =2$. This confirms that lower min_sup$^{\delta }$ thresholds substantially increase mining complexity by expanding the set of candidate FT frequent patterns. Furthermore, the effect of lowering min_sup$^{\delta }$ is significantly more pronounced for *VB-FT-Mine* and *FT-MFI-MultiTree*. In *BMSWebView1* (Table 13), VB-FT-Mine exceeds the 3,200 seconds time limit for most configurations when $\delta =2$, while FT-MFI-MultiTree remains slower than FT-MFI-PG due to repeated construction and traversal of multiple trees. These results reinforce the conclusions drawn from Figs. 7, 8, 9, where both baseline methods struggle at low thresholds.

**Effect of item**_sup$^{\delta }$: The threshold item_sup$^{\delta }$ serves as an additional reliability constraint in the FT itemset mining by ensuring that tolerance does not lead to unreliable patterns. Specifically, although an itemset *X* may satisfy the global FT frequency requirement (i.e., it appears in at least min_sup$^{\delta }$ FT transactions), the FT definition further requires that *each individual item* in *X* must occur in at least item_sup$^{\delta }$ FT transactions of *X*. This condition prevents weak items from being included in an FT frequent itemset simply because $\delta$ allows partial matching. Hence, item_sup$^{\delta }$ acts as an item-level support constraint that strengthens the validity of the discovered FT-MFIs. From a computational perspective, item_sup$^{\delta }$ also strongly affects the search space. A lower item_sup$^{\delta }$ makes the item-level constraint less restrictive, allowing more items to remain eligible for extension during mining. This increases the number of candidate FT frequent itemsets and enlarges the conditional search space, which results in higher processing time. In contrast, a higher item_sup$^{\delta }$ eliminates unreliable items early and reduces the number of candidate extensions explored, leading to smaller FT-FP trees (or conditional trees) and improved efficiency.

Figures 6, 10, and 11 summarize the execution times on the *Retail* dataset under varying values of item_sup$^{\delta }$ while keeping min_sup$^{\delta }$ fixed. The results clearly demonstrate that item_sup$^{\delta }$ has a significant impact on both the runtime and the overall mining complexity. In particular, when item_sup$^{\delta }$ is set to a lower value, more items satisfy the item-level FT frequency constraint and remain eligible for extension during mining. This substantially increases the number of conditional patterns and candidate FT itemsets, resulting in higher execution times for all algorithms. In contrast, increasing item_sup$^{\delta }$ strengthens the pruning effect by eliminating weak items early, which reduces the size of the conditional search space and improves efficiency. Across all tested configurations, *FT-MFI-PG* consistently mines the complete set of FT-MFIs in considerably less time compared to *VB-FT-Mine* and *FT-MFI-MultiTree*. Furthermore, *FT-MFI-PG* demonstrates strong scalability when the dataset size increases (Fig. 11) and when the average transaction length becomes larger (Fig. 10). This indicates that the proposed FP-tree mapping and pattern-growth strategy is able to exploit the pruning effect introduced by higher item_sup$^{\delta }$ thresholds more effectively than the competing approaches. On the other hand, *VB-FT-Mine* and *FT-MFI-MultiTree* show a steep increase in runtime at lower item_sup$^{\delta }$ thresholds and frequently fail to complete the mining process within the 3,200 seconds time limit, since the relaxed item-level constraint leads to a rapid expansion of the candidate space.

Figures 7, 8, and 9 report the execution times observed on the *T10I4D100K*, *FoodMart*, and *BMSWebView1* datasets, respectively, under varying item_sup$^{\delta }$ values. Similar performance trends are observed across these datasets. As item_sup$^{\delta }$ decreases, all algorithms experience increased runtime due to the larger number of frequent items retained and the corresponding growth in the number of candidate FT frequent itemsets. This effect is particularly severe for *VB-FT-Mine*, which relies on Apriori-based candidate generation and therefore suffers from excessive FT support verification when many frequent items exist. Although *FT-MFI-MultiTree* performs better than *VB-FT-Mine* by adopting a pattern-growth paradigm, it still incurs substantial overhead due to repeated construction and traversal of multiple conditional trees. In contrast, *FT-MFI-PG* remains the most efficient across all item_sup$^{\delta }$ configurations, confirming that its compact FP-tree representation and effective pruning strategies allow it to scale well even when item_sup$^{\delta }$ is low and the search space becomes significantly larger (Figs. 12, 13).

**Fig. 14:**
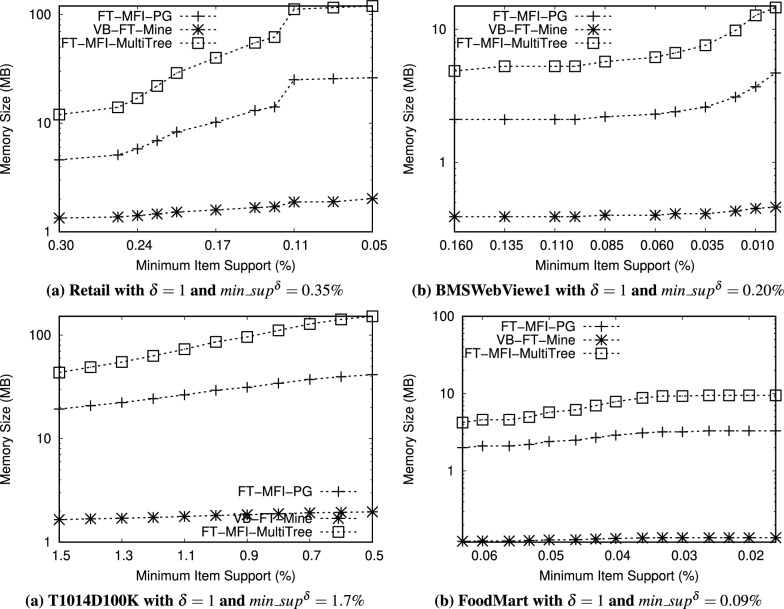
Memory consumption of FT-MFI mining algorithms on different datasets.

*Memory consumption:* Figure 14 compares the memory consumption of the algorithms during execution. Among them, *VB-FT-Mine* uses the least memory. This efficiency is primarily attributed to its bit-vector representation of transactions, where each item is encoded as a binary vector indicating its occurrence across all transactions. Through this encoding, multiple transactions are compressed within a single bit-vector element, significantly reducing storage overhead and minimizing data duplication during computation. Because all subsequent operations such as intersection or union of transactions are performed using lightweight bitwise operations, *VB-FT-Mine* maintains compact memory usage even on large datasets. In contrast, *FT-MFI-PG* consumes more memory than both *VB-FT-Mine*. This is mainly due to its use of the FP-tree structure for storing transactions. Each transaction contributes one or more nodes to the FP-tree, and additional memory is required to maintain pointers linking parent and child nodes. While this representation is efficient for compressing transactions with common prefixes, the node-based structure inherently introduces extra memory overhead compared to the compact bit-vector format. Finally, *FT-MFI-MultiTree* exhibits the highest memory usage among all compared algorithms. This method constructs multiple FP-trees to manage transactions with different FT mismatches. When transactions share a common prefix of items but differ in their allowed tolerance level, they are placed in separate FP-trees rather than being merged. This results in a significant duplication of structures, as redundant nodes and links must be created for similar transactions across different trees. For instance, when mining supersets of (*ab*) with $\delta = 1$, four FP-trees are required: one for transactions containing both *a* and *b*, one for those containing *a* but not *b*, one for those containing *b* but not *a*, and one for those missing both. This replication considerably increases memory consumption and reduces scalability compared to the proposed approach.

**Table 16:** Component analysis of FT–MFI–PG on benchmark datasets (execution time in seconds).

Configuration	$\delta = 1$	$\delta = 2$
Retail ($min\_sup^{\delta }$ = 0.35%, $item\_sup^{\delta }$ = 0.06%)		
FT–MFI–PG – (all components)	26	178
FT–MFI–PG – (FHUT)	21	145
FT–MFI–PG – (HUTMFI)	22	149
FT–MFI–PG – (Max-FT-tree)	23	162
FT–MFI–PG + (all components)	18	127
BMSWebView1 ($min\_sup^{\delta }$ = 0.20%, $item\_sup^{\delta }$ = 0.02%)		
FT–MFI–PG – (all components)	87	832
FT–MFI–PG – (FHUT)	67	769
FT–MFI–PG – (HUTMFI)	72	778
FT–MFI–PG – (Max-FT-tree)	80	824
FT–MFI–PG + (all components)	65	763
T10I4D100K ($min\_sup^{\delta }$ = 1.7%, $item\_sup^{\delta }$ = 0.6%)		
FT–MFI–PG – (all components)	104	3356
FT–MFI–PG – (FHUT)	92	3096
FT–MFI–PG – (HUTMFI)	95	3157
FT–MFI–PG – (Max-FT-tree)	98	3290
FT–MFI–PG + (all components)	84	2985
FoodMart ($min\_sup^{\delta }$ = 0.09%, $item\_sup^{\delta }$ = 0.02%)		
FT–MFI–PG – (all components)	1	102
FT–MFI–PG – (FHUT)	1	88
FT–MFI–PG – (HUTMFI)	1	89
FT–MFI–PG – (Max-FT-tree)	1	98
FT–MFI–PG + (all components)	1	87

### Algorithmic component analysis

The results in Table 16 present a detailed component analysis of the *FT-MFI-PG* algorithm, where execution time is measured in seconds for different datasets. Each configuration selectively removes or combines pruning strategies, thereby illustrating their individual and collective contributions to computational efficiency. The pruning components consist of FHUT and HUTMFI, which discard infrequent FT-MFIs and their supersets at an early stage, while the Max-FT-tree facilitates efficient maximal superset checking. The baseline “FT–MFI–PG – (all components)” corresponds to a simple traversal with no pruning, whereas “FT–MFI–PG + (all components)” represents the complete configuration with all pruning enabled.

Across all datasets, the inclusion of pruning consistently reduces execution time, confirming the effectiveness of these strategies. Both FHUT and HUTMFI yield substantial savings, with HUTMFI slightly outperforming FHUT, reflecting its more efficient superset lookups. The Max-FT-tree also contributes noticeably to reducing execution times by accelerating maximality verification. The combination of all components provides the largest savings, highlighting the complementary nature of these pruning strategies. When comparing dataset characteristics, a clear difference emerges between less sparse datasets (Retail and T10I4D100K) and more sparse datasets (BMSWebView1 and FoodMart). On less sparse datasets, where the number of potential itemsets and supersets is considerably higher, pruning has a much larger impact. For example, on T10I4D100K at $\delta = 2$, the execution time decreases from 3356 seconds with no pruning to 2985 seconds with all pruning components. Similarly, for the Retail dataset, the execution time reduces from 178 to 127 seconds. These improvements demonstrate that pruning is especially critical in dense settings where the search space grows exponentially. In contrast, the benefits of pruning are less pronounced on more sparse datasets. On FoodMart, the execution time at $\delta = 2$ decreases from 102 seconds without pruning to 87 seconds with all pruning. The smaller gain can be explained by the inherently limited search space of sparse datasets, where fewer frequent itemsets exist and pruning opportunities are reduced. A similar trend is observed on BMSWebView1, where pruning improves execution time but with a smaller relative impact compared to dense datasets. Overall, the component analysis demonstrates that while all pruning components contribute to performance improvement, their impact is magnified in less sparse datasets with large numbers of frequent patterns.

## Conclusion

In this paper, we introduce a new algorithm, *FT-MFI-PG*, that mines maximal fault-tolerant (FT) frequent itemsets using pattern-growth approach. The *FT-MFI-PG* algorithm provides several key improvements over traditional Apriori-based FT-MFI mining algorithms. First, it organizes FT transactions into a compact data structure known as the FT-FP-tree. By mapping transactions with similar itemsets along shared branches, this structure greatly reduces the effective size of the dataset during mining, which in turn allows for more efficient computation of itemset supports. Second, unlike Apriori-style approaches that generate all possible candidate itemsets level by level, *FT-MFI-PG* employs a pattern-growth strategy to generate candidates itemsets directly from the conditional patterns of the FT-FP-tree. This targeted generation ensures that only itemsets present in the dataset are considered, substantially lowering the total number of candidate itemsets and improving overall computational efficiency. We conducted a series of experiments to evaluate the performance of *FT-MFI-PG* in comparison to existing FT-MFI mining algorithms across multiple benchmark datasets with diverse sizes and transaction characteristics. The experimental results indicate that *FT-MFI-PG* outperforms existing FT-MFI mining algorithms, efficiently mining the complete set of FT-MFIs while scaling effectively to handle spare and dense datasets.

Although the proposed *FT-MFI-PG* algorithm demonstrates significant efficiency and scalability, its FP-tree–based structure is still subject to the well-known worst-case memory overhead of FP-trees. In particular, when transactions share very few common prefixes or when the dataset exhibits extremely high dimensionality with uniformly distributed items, the FP-tree may degenerate into a large, nearly uncompressed structure in which most branches are unique. Under such conditions, the FT-FP-tree and its associated FT conditional pattern tables may consume significantly more memory than in typical, prefix-rich datasets. This limitation is inherent to all FP-growth based approaches; therefore, while the proposed algorithm remains more memory efficient than prior FT pattern-growth algorithm that build multiple FP-trees, it may still experience elevated memory usage in datasets with minimal prefix sharing.

Furthermore, The current algorithm is designed for static transactional datasets and does not yet support incremental or real-time mining of fault-tolerant itemsets in dynamic data streams. Future extensions may focus on adaptive mechanisms for automatic $\delta$ adjustment based on observed data noise levels or evolving transaction characteristics. Furthermore, implementing parallel or distributed versions of *FT-MFI-PG* using multi-core or cluster based architectures could further improve performance for very large datasets. Additional research directions include mining the top-*k* FT frequent itemsets without relying on a minimum support threshold or discovering closed FT frequent itemsets to reduce redundancy.

## Data Availability

The datasets used in this study are publicly available from the Frequent Itemset Mining Dataset Repository (FIMI) at https://fimi.uantwerpen.be/data/

## References

[1] Luna, J. M., Fournier-Viger, P. & Ventura, S. Frequent itemset mining: A 25 years review. *Wiley Interdiscip. Rev. Data Min. Knowl. Discov.* **9**, e1329 (2019).

[2] Chee, C.-H., Jaafar, J., Aziz, I. A., Hasan, M. H. & Yeoh, W. Algorithms for frequent itemset mining: A literature review. *Artif. Intell. Rev.* **52**, 2603–2621 (2019).

[3] Huang, Q., Luo, P. & Tung, A. K. A new sparse data clustering method based on frequent items. *Proc. ACM Manag. Data* **1**, 1–28 (2023).

[4] Fung, B. C. M., Wang, K. & Ester, M. Hierarchical document clustering using frequent itemsets. In *Proceedings of the 3rd SIAM International Conference on Data Mining, San Francisco, CA, USA, 2003*, 59–70, 10.1137/1.9781611972733.6 (2003).

[5] Zhang, W., Yoshida, T., Tang, X. & Wang, Q. Text clustering using frequent itemsets. *Knowl.-Based Syst.* **23**, 379–388 (2010).

[6] Jiang, F., Leung, C. K. & Zhang, H. B-mine: frequent pattern mining and its application to knowledge discovery from social networks. In *Proceedings of the Asia-Pacific Web Conference, 2016*, 316–328 (Springer, 2016).

[7] Moosavi, S. A., Jalali, M., Misaghian, N., Shamshirband, S. & Anisi, M. H. Community detection in social networks using user frequent pattern mining. *Knowl. Inf. Syst.* **51**, 159–186. 10.1007/s10115-016-0970-8 (2017).

[8] Farzanyar, Z. & Cercone, N. Efficient mining of frequent itemsets in social network data based on mapreduce framework. In *Proceedings of the IEEE/ACM International Conference on Advances in Social Networks Analysis and Mining, 2013* 1183–1188 (2013).

[9] Han, J., Cheng, H., Xin, D. & Yan, X. Frequent pattern mining: Current status and future directions. *Data Min. Knowl. Disc.* **15**, 55–86 (2007).

[10] Çatkın, M., Yüksel, Ş., Konyalıoğlu, A. K., Apaydın, T. & Özcan, T. Market basket analysis using apriori and eclat algorithm in an e-commerce company. In *Proceedings of the International Conference on Intelligent and Fuzzy Systems, 2025*, 745–754 (Springer, 2025).

[11] Tripathi, D., Nigam, B. & Edla, D. R. A novel web fraud detection technique using association rule mining. *Procedia Comput. Sci.* **115**, 274–281 (2017).

[12] Ngo, M.-H., Do, H.-H., Nguyen, H.-S., Pham, H.-D. & Do, Q.-H. Anomaly detection based on frequent pattern mining in smart home devices. *IEEE Access* (2025).

[13] Seeja, K. & Zareapoor, M. Fraudminer: A novel credit card fraud detection model based on frequent itemset mining. *Sci. World J.* **2014**, 252797 (2014). 10.1155/2014/252797 PMC4180893 25302317

[14] Creighton, C. & Hanash, S. Mining gene expression databases for association rules. *Bioinformatics* **19**, 79–86 (2003). 12499296 10.1093/bioinformatics/19.1.79

[15] Lee, G., Peng, S.-L. & Lin, Y.-T. Proportional fault-tolerant data mining with applications to bioinformatics. *Inf. Syst. Front.* **11**, 461–469. 10.1007/s10796-009-9158-z (2009). 32214877 10.1007/s10796-009-9158-z PMC7087812

[16] Mallik, S., Mukhopadhyay, A. & Maulik, U. Ranwar: Rank-based weighted association rule mining from gene expression and methylation data. *IEEE Trans. NanoBioscience* **14** (2015). 10.1109/TNB.2014.2359494 25265613

[17] Hundekari, S. et al. Efficient frequent subgraph mining: Algorithms and applications in complex networks. In *Graph Mining: Practical Uses and Instruments for Exploring Complex Networks*, 55–65 (Springer, 2025).

[18] Iváncsy, R. & Vajk, I. Frequent pattern mining in web log data. *Acta Polytechnica Hungarica* **3** (2006).

[19] Yu, X. & Korkmaz, T. Heavy path based super-sequence frequent pattern mining on web log dataset. *Artif. Intell. Res.* **4**, 1–12. 10.5430/air.v4n2p1 (2015).

[20] Kavitha, D. & Kalpana, B. Frequent itemset generation using enhanced fuzzy apriori algorithm for web log session data. In *Proceedings of the International Conference on Computer Communication and Informatics (ICCCI), 2017*, 1–9 (IEEE, 2017).

[21] Agrawal, R. & Srikant, R. Fast algorithms for mining association rules. In *Proceedings of the 20th International Conference on VLDB, 1994*, 487–499 (1994).

[22] Han, J., Pei, J. & Yin, Y. Mining frequent patterns without candidate generation. In *Proceedings of the 2000 ACM SIGMOD International Conference on Management of Data, May 16-18, Dallas, Texas, USA, 2000*, 1–12, 10.1145/342009.335372 (2000).

[23] Kosters, W. A. & Pijls, W. Apriori, A depth first implementation. In *FIMI ’03, Frequent Itemset Mining Implementations, Proceedings of the ICDM 2003 Workshop on Frequent Itemset Mining Implementations, 19 December, Melbourne, Florida, USA, 2003* (2003).

[24] Bodon, F. A fast APRIORI implementation. In *FIMI ’03, Frequent Itemset Mining Implementations, Proceedings of the ICDM 2003 Workshop on Frequent Itemset Mining Implementations, 19 December, Melbourne, Florida, USA, 2003* (2003).

[25] Liu, G., Lu, H., Yu, J. X., Wang, W. & Xiao, X. AFOPT: an efficient implementation of pattern growth approach. In *FIMI ’03, Frequent Itemset Mining Implementations, Proceedings of the ICDM 2003 Workshop on Frequent Itemset Mining Implementations, 19 December, Melbourne, Florida, USA, 2003* (2003).

[26] Uno, T., Kiyomi, M. & Arimura, H. LCM ver. 2: Efficient mining algorithms for frequent/closed/maximal itemsets. In *Proceedings of the IEEE ICDM Workshop on Frequent Itemset Mining Implementations, Brighton, UK, 1st November, 2004* (2004).

[27] Burdick, D., Calimlim, M., Flannick, J., Gehrke, J. & Yiu, T. MAFIA: A maximal frequent itemset algorithm. *IEEE Trans. Knowl. Data Eng.* **17**, 1490–1504. 10.1109/TKDE.2005.183 (2005).

[28] Vo, B., Pham, S., Le, T. & Deng, Z. A novel approach for mining maximal frequent patterns. *Expert Syst. Appl.* **73**, 178–186. 10.1016/j.eswa.2016.12.023 (2017).

[29] Gan, W., Lin, J. C., Fournier-Viger, P., Chao, H. & Zhan, J. Mining of frequent patterns with multiple minimum supports. *Eng. Appl. Artif. Intell.* **60**, 83–96. 10.1016/j.engappai.2017.01.009 (2017).

[30] Chen, R., Zhao, S. & Liu, M. A fast approach for up-scaling frequent itemsets. *IEEE Access* **8**, 97141–97151 (2020).

[31] Chen, S., Nie, L., Tao, X., Li, Z. & Zhao, L. Approximation of probabilistic maximal frequent itemset mining over uncertain sensed data. *IEEE Access* **8**, 97529–97539 (2020).

[32] Al-Bana, M. R., Farhan, M. S. & Othman, N. A. An efficient spark-based hybrid frequent itemset mining algorithm for big data. *Data* **7**, 11 (2022).

[33] Saikaew, K. R., Leung, C. K. & Phiwhorm, K. An enhanced fp-growth algorithm with hybrid adaptive support threshold for association rule mining. In *Proceedings of the International Conference on Big Data Analytics and Knowledge Discovery, 2025*, 102–108 (Springer, 2025).

[34] Jeya Sutha, M. & Ramesh Dhanaseelan, F. A novel multiprocessing-based closed frequent itemset mining algorithm for the detection of cardiovascular disease. *IETE J. Res.* 1–14 (2025).

[35] Pei, J., Tung, A. K. H. & Han, J. Fault-tolerant frequent pattern mining: Problems and challenges. In *Proceedings of the ACM SIGMOD Workshop on Research Issues in Data Mining and Knowledge Discovery, Santa Barbara, CA, USA, 20th May, 2001* (2001).

[36] Cheng, H., Yu, P. S. & Han, J. Approximate frequent itemset mining in the presence of random noise. In *Soft Computing for Knowledge Discovery and Data Mining*, 363–389 (Springer, 2008).

[37] Yu, X., Li, Y. & Wang, H. Mining approximate frequent patterns from noisy databases. In *Proceedings of the 10th International Conference on Broadband and Wireless Computing, Communication and Applications (BWCCA), 2015* (2015).

[38] Cui, Y., Gan, W., Lin, H. & Zheng, W. Fri-miner: Fuzzy rare itemset mining. *Appl. Intell.* **52**, 3387–3402 (2022).

[39] Cheung, Y.-L. & Fu, A.W.-C. Mining frequent itemsets without support threshold: With and without item constraints. *IEEE Trans. Knowl. Data Eng.* **16**, 1052–1069. 10.1109/TKDE.2004.44 (2004).

[40] Huynh-Thi-Le, Q., Le, T., Vo, B. & Le, B. An efficient and effective algorithm for mining top-rank-k frequent patterns. *Expert Syst. Appl.* **42**, 156–164. 10.1016/j.eswa.2014.07.045 (2015).

[41] Saif-Ur-Rehman, Ashraf, J., Habib, A. & Salam, A. Top-k miner: Top-k identical frequent itemsets discovery without user support threshold. *Knowl. Inf. Syst.* **48**, 741–762, 10.1007/s10115-015-0907-7 (2016).

[42] Lin, J. & Li, Y. Finding approximate frequent patterns in streaming medical data. In *Proceedings of the 23rd IEEE International Symposium on Computer-Based Medical Systems (CBMS), 2010*, 13–18 (IEEE, 2010).

[43] Silvestri, C. & Orlando, S. Approximate mining of frequent patterns on streams. *Intell. Data Anal.* **11**, 49–73 (2007).

[44] Ashraf, S. A. & Nafis, M. T. Fault tolerant frequent patterns mining in large datasets having certain and uncertain records. *Adv. Comput. Sci. Technol.* **10**, 2381–2394 (2017).

[45] Bashir, S. & Baig, A. R. Max-ftp: Mining maximal fault-tolerant frequent patterns from databases. In *Proceedings of the 24th British National Conference on Databases BNCOD, Glasgow, UK, July 3-5, 2007*, vol. 4587 of *Lecture Notes in Computer Science*, 235–246, 10.1007/978-3-540-73390-4_26 (Springer, 2007).

[46] Liu, S. & Poon, C. K. On mining approximate and exact fault-tolerant frequent itemsets. *Knowl. Inf. Syst.* **55**, 361–391. 10.1007/s10115-017-1079-4 (2018).

[47] Bashir, S. An efficient pattern growth approach for mining fault tolerant frequent itemsets. *Expert Syst. Appl.* **143**, 113046 (2020). 32288329 10.1016/j.eswa.2019.113046 PMC7126664

[48] Koh, J. & Yo, P. An efficient approach for mining fault-tolerant frequent patterns based on bit vector representations. In *Proceedings of the 10th Database Systems for Advanced Applications (DASFAA), Beijing, China, April 17-20, 2005*, 568–575, 10.1007/11408079_51 (2005).

[49] Han, J., Pei, J., Yin, Y. & Mao, R. Mining frequent patterns without candidate generation: A frequent-pattern tree approach. *Data Min. Knowl. Disc.* **8**, 53–87. 10.1023/B:DAMI.0000005258.31418.83 (2004).

[50] Han, J. & Pei, J. Pattern-growth methods. In *Frequent Pattern Mining*, 65–81, 10.1007/978-3-319-07821-2_3 (2014).

[51] Bashir, S., Halim, Z. & Baig, A. R. Mining fault tolerant frequent patterns using pattern growth approach. In *Proceedings of the 6th ACS/IEEE International Conference on Computer Systems and Applications, Doha, Qatar, March 31 - April 4, 2008*, 172–179, 10.1109/AICCSA.2008.4493532 (2008).

[52] Gouda, K. & Zaki, M. J. Efficiently mining maximal frequent itemsets. In *Proceedings of the IEEE International Conference on Data Mining, 2001*, 163–170 (IEEE, 2001).

[53] Lee, G. & Lin, Y.-T. A study on proportional fault-tolerant data mining. In *Proceedings of the 2006 International Conference on Innovations in Information Technology, Dubai, 2006* (2006).

[54] Baek, Y. et al. Approximate high utility itemset mining in noisy environments. *Knowl. Based Syst.* **212**, 106596 (2021).

[55] Liu, S. & Poon, C. K. On mining proportional fault-tolerant frequent itemsets. In *Proceedings of the International Conference on Database Systems for Advanced Applications, 2014* 342–356. 10.1007/978-3-319-05810-8_23 (2014).

[56] Zoraghchian, A. A., Sohrabi, M. K. & Yaghmaee, F. Parallel frequent itemsets mining using distributed graphic processing units. *Multimed. Tools Appl.* **81**, 43873–43895 (2022).

[57] Zhang, J. et al. Remaining useful life prediction based on self-attention mechanism-sequential variational autoencoder: From a semi-supervised perspective. *Adv. Eng. Inform.* **71**, 104242 (2026).

[58] Zhang, J. et al. A transferable topology-aware graph pooling network for remaining useful life prediction under cross-domain conditions. *IEEE Trans. Reliab.* (2025).

[59] Jiang, H. & Meng, H. A parallel fp-growth algorithm based on gpu. In *Proceedings of the IEEE 14th International Conference on e-Business Engineering (ICEBE), 2017*, 97–102 (IEEE, 2017).

[60] Chon, K.-W., Hwang, S.-H. & Kim, M.-S. Gminer: A fast gpu-based frequent itemset mining method for large-scale data. *Inf. Sci.* **439**, 19–38 (2018).

[61] Chon, K.-W. & Kim, C. Gminer++: Boosting gpu-based frequent itemset mining by reducing redundant computations. *Expert Syst. Appl.* **250**, 123928 (2024).

[62] Gouda, K. & Zaki, M. J. Genmax: An efficient algorithm for mining maximal frequent itemsets. *Data Min. Knowl. Disc.* **11**, 223–242 (2005).

[63] Grahne, G. & Zhu, J. High performance mining of maximal frequent itemsets. In *Proceedings of the 6th International Workshop on High Performance Data Mining, 2003*, Vol. 16, 34 (2003).

[64] Bayardo, R. J. J. Efficiently mining long patterns from databases. In *Proceedings of the ACM SIGMOD International Conference on Management of Data, June 2-4, Seattle, Washington, USA, 1998*, 85–93, 10.1145/276304.276313 (ACM Press, 1998).

[65] Bashir, S. & Baig, A. Fastlmfi: An efficient approach for local maximal patterns propagation and maximal patterns superset checking. In *Proceedings of the IEEE International Conference on Computer Systems and Applications, 2006*, 452–459 (IEEE, 2006).

